# Overexpression of ATPase Na+/K+ transporting alpha 1 polypeptide, ATP1A1, correlates with clinical diagnosis and progression of esophageal squamous cell carcinoma

**DOI:** 10.18632/oncotarget.13267

**Published:** 2016-11-10

**Authors:** I-Chen Wu, Yu-Kuei Chen, Chun-Chieh Wu, Yu-Jen Cheng, Wei-Chung Chen, Huey-Jiun Ko, Yu-Peng Liu, Chee-Yin Chai, Hung-Shun Lin, Deng-Chyang Wu, Ming-Tsang Wu

**Affiliations:** ^1^ Division of Gastroenterology, Department of Internal Medicine, Kaohsiung Medical University Hospital, Kaohsiung, Taiwan; ^2^ Department of Medicine, Faculty of Medicine, College of Medicine, Kaohsiung Medical University, Kaohsiung, Taiwan; ^3^ Department of Food Science and Nutrition, Meiho University, Pingtung, Taiwan; ^4^ Department of Pathology, Kaohsiung Medical University Hospital, Kaohsiung, Taiwan; ^5^ Department of Surgery, E-Da Hospital, Kaohsiung, Taiwan; ^6^ Ph.D. Program in Environmental and Occupational Medicine, Kaohsiung Medical University Hospital, Kaohsiung, Taiwan; ^7^ Graduate Institute of Clinical Medicine, Kaohsiung Medical University, Kaohsiung, Taiwan; ^8^ Center for Infectious Disease and Cancer Research, Kaohsiung Medical University, Kaohsiung, Taiwan; ^9^ Department of Laboratory Medicine & Department of Research, Education & Training, Kaohsiung Municipal Hsiao-Kang Hospital, Kaohsiung Medical University, Kaohsiung, Taiwan; ^10^ Department of Family Medicine, Kaohsiung Medical University Hospital, Kaohsiung, Taiwan; ^11^ Research Center for Environmental Medicine, Kaohsiung Medical University, Kaohsiung, Taiwan

**Keywords:** ATPase Na+/K+ transporting alpha 1 polypeptide, microarray-based screening, arecoline, F344, esophageal squamous cell carcinoma

## Abstract

This study aims to identify new upregulated genes related to secretory or membranous proteins to help detect esophageal squamous cell carcinoma (ESCC). First, we performed microarray-based screening of esophageal tumors from both N-nitrosomethylbenzylamine- and arecoline-induced F344 rats and seventeen human ESCC specimens. Candidate genes were validated by quantitative PCR (qPCR) and immunohistochemical (IHC) staining of ESCC tissues. Among the paired cancer and adjacent normal tissues from 14 ESCC patients, 10 pairs (71.4%) had overexpression of ATP1A1 (ATPase Na+/K+ transporting alpha 1 polypeptide) by qPCR (P = 0.0052). ATP1A1 protein expression was re-confirmed by tissue arrays in 243 ESCC tissues and 126 adjacent normal tissues and by ELISA in 78 serum specimens of ESCC patients. ATP1A1 was 12.3 times (adjusted odds ratio=12.3, 95% CI = 7.2-21.0) more likely to be overexpressed in cancer tissues than in normal tissues. ATP1A1 expression was also correlated to tumor stage. Patients with higher serum ATP1A1 levels had a 2.9-fold (95% CI = 1.1-7.4) risk of late-stage disease (stages III-IV *vs*. I-II). Downregulation of ATP1A1 expression inhibited the migration and invasion ability of ESCC cell lines *in vitro*. We concluded that the overexpression of ATP1A1 is strongly associated with the presence and severity of ESCC.

## INTRODUCTION

Esophageal cancer is the sixth leading cause of cancer death and the eighth most common cancer worldwide [1]. Squamous cell carcinoma (ESCC) is still the predominant histological type of this cancer in most parts of the world, including Taiwan [1, 2]. By the time that patients develop symptoms such as dysphagia, they usually have advanced diseases and poor prognosis, the 5-year survival rate ranging from 15-25% [1]. Thus, it is crucial to search for novel markers to assist in the early detection of ESCC and help predict outcome.

Areca nut chewing is the fourth most popular substance abused in the world [3]. About six hundred million people chew areca nut around the world, especially in India, Southeast Asia, and Taiwan [2, 4]. Previous epidemiological studies, including ours, have found areca nut chewing to be an independent risk factor for ESCC [2].

Fischer 344 (F344) rats are commonly used to study esophageal tumorigenesis induced by N-nitrosomethylbenzylamine (NMBA) *in-vivo* [5]. One of the major alkaloids of areca nut, arecoline, has been shown to cause strong genotoxicity and is considered a potential carcinogen [6, 7]. However, whether adding arecoline in a NMBA-induced F344 model can promote the carcinogenesis of ESCC is unclear. In this study, we first applied microarray-based screening to both NMBA- and arecoline-induced esophageal tumors in F344 rats as well as human ESCC specimens to search for novel genes encoding secretory or membranous proteins that might potentially be used to diagnose ESCC and track it progression. ATPase Na+/K+ transporting alpha 1 polypeptide (ATP1A1) was found to be the best candidate gene for this purpose.

ATP1A1 is a transmembrane protein that catalyzes the active transport of Na^+^ and K^+^ and maintains intracellular ion homeostasis of epithelial cells. Emerging evidence suggests that iron channels and pumps play a role in tumor development and progression, suggesting cancer is also a disease of “channelopathy” [8]. In addition to exchanging cations, ATP1A1 is also involved in the tumorigenesis and migration of cancer cells [9–11]. Overexpression of ATP1A1 has been reported in tumor specimens from patients with medulloblastoma [12], glioblastoma [13], melanoma [14], hepatomas [15], and non-small-cell lung cancer [16]. In contrast, down-regulation of ATP1A1 has been found in colorectal cancer [17]. To the best of our knowledge, no study has investigated ATP1A1's role in esophageal cancer. We performed *in-vitro*, *in-vivo*, and clinical studies to investigate its possible role in this deadly and invasive disease.

## RESULTS

### Papilloma in the esophagus

**Figure 1:**
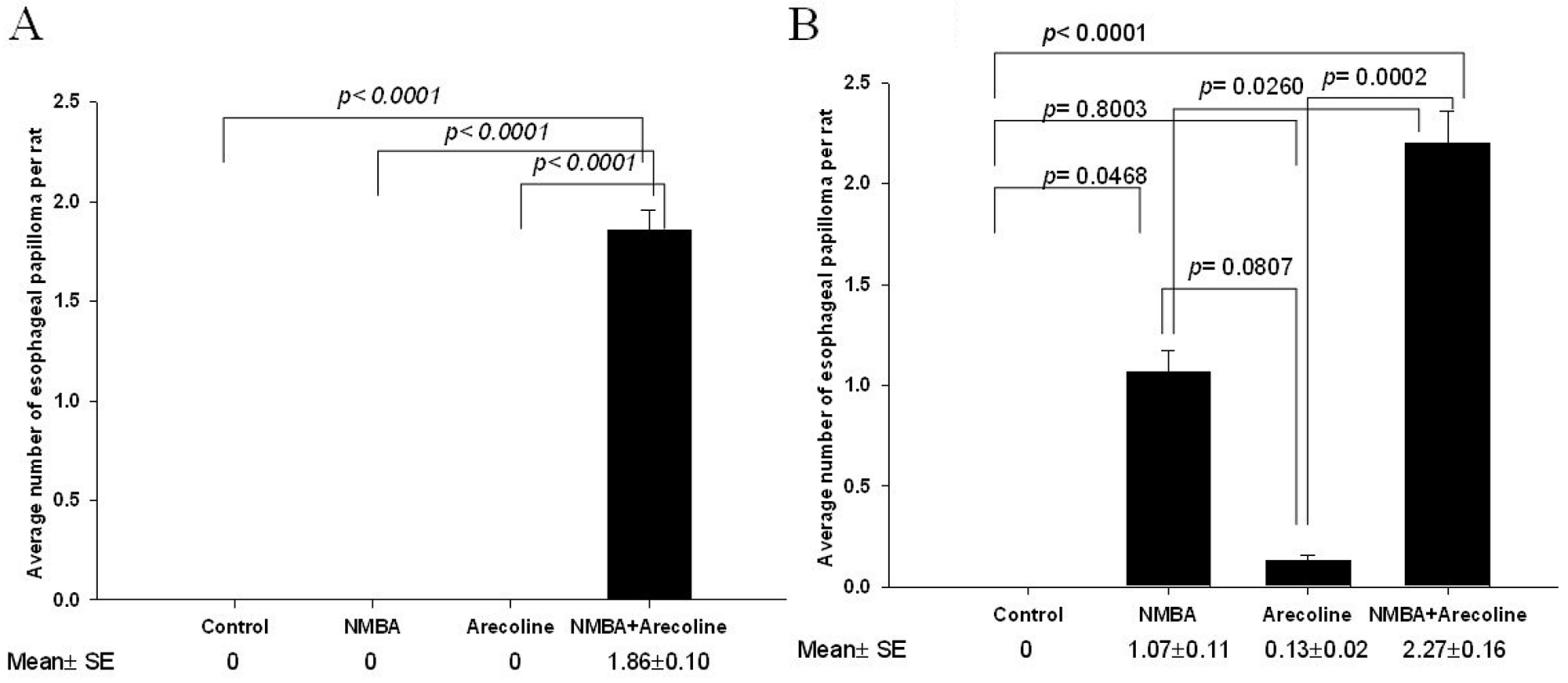
Murine experiments in F344 rats **A.** Average number of papillomas of the esophagus in the 25th weeks; **B.** in the 30th weeks. The data were analyzed using one-way ANOVA and significance was assessed by the LSD post hoc test.

By the 25th week, all seven rats belonging to the NMBA + Arecoline group developed different numbers of esophageal papilloma, ranging from one to three (Supplementary Table S1 and additional information on Supplementary Materials), while no papilloma was found in the other three groups. The averaged number (± SE) of esophageal papilloma per rat was 1.86 ± 0.10 in the NMBA + Arecoline group, which was significantly more papillomas than was found in the other three groups (*p* < 0.0001) (Figure 1A).

By the 30th week, 2 (13.3%), 8 (53.3%), and 11 (73.3%) out of fifteen rats in the Arecoline, the NMBA, and the NMBA + Arecoline groups, respectively, developed esophageal papilloma, while none of the control group had (Supplementary Table S1). The average number (± SE) of esophageal papilloma per rat was 0 in the control group, 0.13 ± 0.02 in the Arecoline group, 1.07 ± 0.11 in the NMBA group, and 2.27± 0.16 in the NMBA+ Arecoline group (Figure 1B). The NMBA + Arecoline group had significantly more papillomas than the NMBA group (*p* = 0.0260). They also had had significantly more papillomas than the control group (*p* < 0.0001) and the Arecoline group (*p* = 0.0002). Although the NMBA group had more papillomas than the control group (*p* = 0.0468) and the Arecoline group, the difference was not significant (*p* = 0.0807). The Arecoline group and the control had a similar number of papillomas (*p* = 0.8003).

### Histopathological images of the esophageal tumors in rats

The representative gross appearance and histological HE-staining of resected rat esophageal tissues at the end of 25th and 30th weeks are shown in Supplementary Figure S1. The esophagus shows a squamous cell papilloma composed of hyperplastic squamous epithelium arranged in papillary fronds in the NMBA+ Arecoline group and the NMBA group in the 25th week and two squamous cell papillomas in the NMBA+ Arecoline group in the 30th week. No pathological changes were found in the control group at 25 and 30 weeks.

### Identification of up-regulation genes from Rat One array and selection of candidate genes for clinical application

Because only the rats in the NMBA + Arecoline group developed esophageal papilloma in week 25, we pooled all esophageal papilloma and pooled the normal esophageal tissues separately in this NMBA + Arecoline group to create a Rat One array analysis. We also pooled the esophageal tissues from the control group in the 25th week for Rat One array analysis as another control. Each pooled sample of these three esophageal normal and tumor tissue microarray assays was performed in duplicate, creating a need for six chips in this experiment. Supplementary Figure S2 shows the gene expressions in the repeated arrays of the three pooled tissue samples were highly correlated (Pearson correlation coefficient r = 0.9848-0.9940).

**Figure 2:**
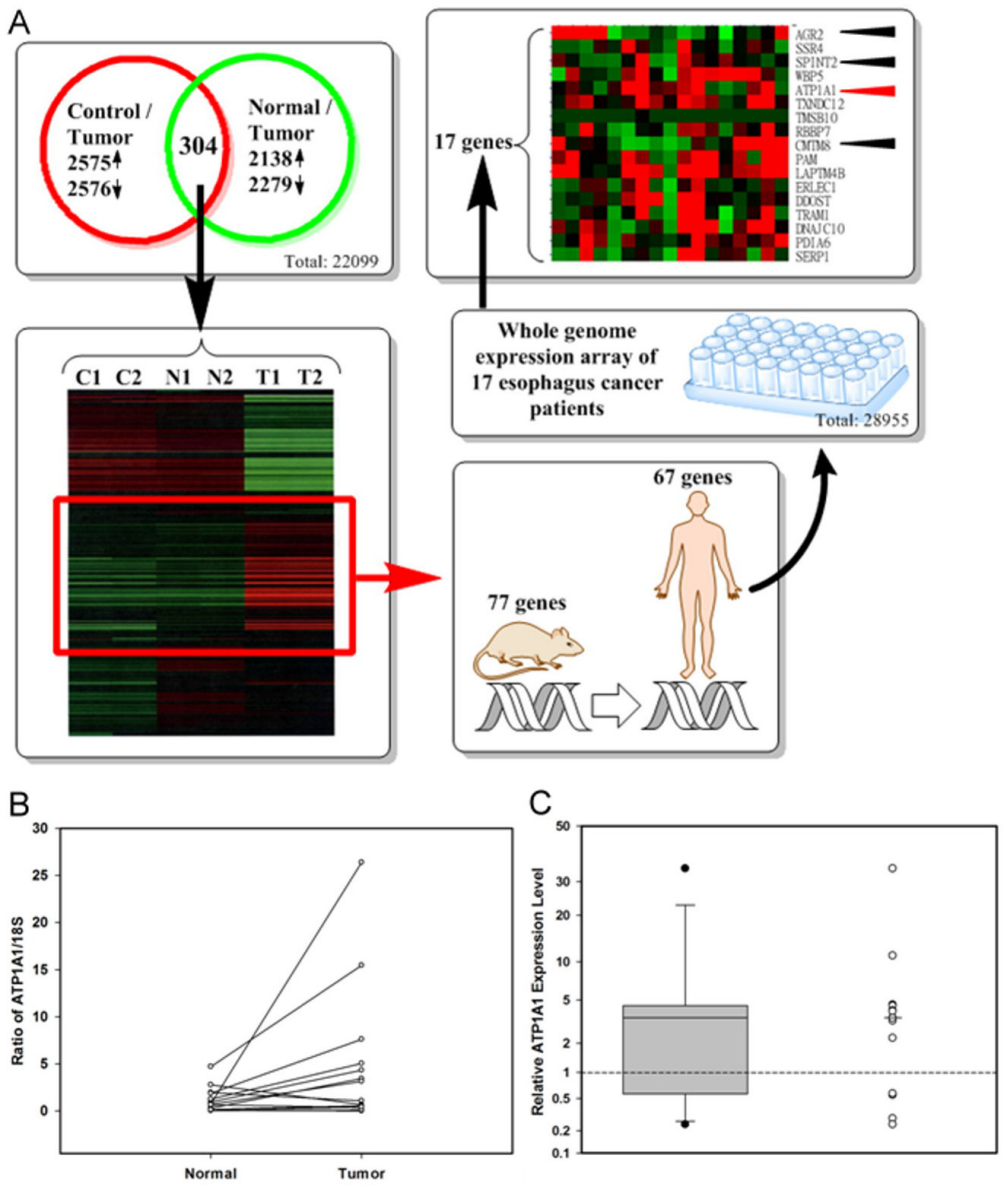
Identification of up-regulation candidate genes from Rat One array and selection of candidate genes for clinical application **A.** The flow chart for identifying candidate genes (Control: DMSO treated only; Normal: normal part from NMBA and arecoline treated rats; Tumor: tumor part from NMBA and arecoline treated rats); **B.** ATP1A1 RNA levels in tumor and adjacent normal tissues from 14 ESCC patients by real-time PCR ((Calculated by 1000 × 2^–(ATP1A1_AverageCT – 18S_AverageCT)^; **C.** The distribution of relative ATP1A1 RNA expression levels (tumor vs. adjacent normal parts) by boxplot and dotplot (Minimum: 0.24, the first quartile: 0.99, median: 3.49; the third quartile: 4.36, and maximum: 34.40). Wilcoxon signed rank test, p = 0.0052.

Genes were selected in steps. First, using “2-fold percentage” less than 15 percent criteria, we found that 22,099 (90.7%) out of 24,358 genes had passed the quality control of Rat One array experiment. Then, we narrowed the selection based on differences in gene expression in tumor parts *vs.* normal parts and tumor parts *vs.* control group, with a log_2_-fold change of ≥ 1 and P-value of < 0.05. Comparing tumor parts with normal parts, we selected 2,138 up-regulated and 2,279 down-regulated genes (Figure 2A). Comparing tumor parts with controls, we selected 2,575 up-regulated and 2,576 down-regulated genes (Figure 2A). Using unsupervised hierarchical clustering analysis with the intensity between the maximum and minimum values exceeding 21,000 to cluster the expression profiles, we found 304 clustering genes, 77 of which were found to be both upregulated in the tumor parts compared to the normal parts and in tumor parts compared to the controls.

Of the 77 candidate rat genes, 67 matched the appropriate human genes (Figure 2A). Seventeen of the 67 candidate genes were also up-regulated in more than half of seventeen paired tissues from seventeen ESCC patients. Four of these genes-ATP1A1 (12/17), SPINT2 (11/17), CMTM8 (10/17), and AGR2 (9/17)-encoded secretory or membrane protein and were chosen. The most consistent expression gene, ATP1A1, in murine model and clinical specimens was further verified by qPCR in paired tumor and adjacent normal tissues of esophagus from 14 ESCC patients (Figure 2B and 2C).We found that ATP1A1 RNA was overexpressed in 10 (71.4%) out of 14 pairs, when tumor parts were compared to their adjacent normal parts (Figure 2B; details in Supplementary Table S2). The median relative ATP1A1 RNA expression level was 3.49 in the 14-paired ESCC samples (Figure 2C). Because the expressions of SPINT2 and CMTM8 in qPCR were not consistent with the microarray data and the expression of AGR2 in IHC staining was not consistent with the microarray data (data not shown), we focused our study on the expression of ATP1A1.

### ATP1A1 expression in esophageal tissues

In total, IHC staining found ATP1A1 to be expressed in 369 ESCC tissue specimens from three different countries (Taiwan, Korea, and USA), which included 126-paired samples with cancer parts and their normal parts and 243 cancer samples only (Table 1, Supplementary Table S3). The mean age of specimen donors ranged from 55-60 yrs and most were male. There were no significant age and gender differences in donors with different IHC scores (Supplementary Table S4).

**Table 1:** Distribution of selected demographic and clinical characteristics of esophageal squamous cell carcinoma patients and their controls

Variables	Controls/ Case 1^1^ (n = 126/126)	Case 2 (n = 243)	Case 3^2^ (n = 319)
	Mean ± SD or n (%)		
Source			
Taiwan	42	-	42
USA	84	189	223
Korea	-	54	54
Age (yrs)	55.7 ± 8.4	58.7 ± 8.4	57.7 ± 8.7
≤ 65	111 (88.1)	188 (77.4)	261 (82.8)
> 65	15 (12.9)	55 (22.6)	58 (18.2)
Gender			
male	96 (76.2)	183 (75.3)	246 (77.1)
female	30 (23.8)	60 (24.7)	73 (22.9)
Clinical staging			
T			
T1			15 (4.7)
T2			67 (21.0)
T3			232 (72.7)
T4			5 (1.6)
N			
N0			226 (70.8)
N1			93 (29.2)
M			
M0			310 (97.2)
M1			9 (2.8)
Stage			
I-II			220 (69.0)
III-IV			99 (31.0)

1 Controls/Cases 1: Adjacent normal part/Tumor part

2 No clinical information of TNM staging in 50 patients.

The overexpression of ATP1A1 was significantly more frequent in tumor parts than their adjacent normal parts in 126-paired samples (*p* < 0.0001; McNemar's test) (Table 2). Categorizing samples into those with IHC score of < 1 and ≥ 1, we found that the significantly higher frequency of ATP1A1 overexpression in cancer tissues than in normal ones after adjusting for age and sex (AOR = 12.3, 95% CI = 7.2-21.0) (Table 2). In addition, the more advanced the clinical stage, the higher the frequency of ATP1A1 overexpression. In the 319 patients with clinical TNM staging information, those with IHC scores ≥ 3 tended to have late stage of ESCC, compared to those with IHC scores < 1 (AOR = 3.6, 95% CI= 1.5-8.7) after adjusting for age and sex (Table 2).

**Table 2:** Relationship between disease and ATP1A1 protein expression in esophageal cancer patients

Variable	Control^1^ (n=126)	Case 1 (n = 126)	Case 2 (n = 243)	Case 3^2^ (n=319)		Crude OR (95% CI)	AOR^2^ (95% CI)
IHC score				Stage I-II (n = 220)	Stage III-IV ( n= 99)		
Risk		n (%)		n (%)			
<1	84 (66.7)	38 (30.2)	36 (14.8)			1.0	1.0
1-<2	42 (33.3)	53 (42.0)	92 (37.9)			5.1 (3.0-8.7)^3^*	5.5 (3.2-9.6)^3^*
2-<3	0 (0)	29 (23.0)	80 (32.9)			-	-
≥3	0 (0)	6 (4.8)	35 (14.4)			-	-
<1	84 (67)	38 (30.2)	36 (14.8)			1.0	1.0
≥1	42 (33)	88 (69.8)	207 (85.2)			11.5 (6.9-19.2)^3^*	12.3 (7.2-21.0)^3^*
Progression							
<1				54 (24.6)	14 (14.1)	1.0	1.0
1-<2				85 (38.6)	35 (35.4)	1.6 (0.8-3.2)	1.7 (0.8-3.4)
2-<3				61 (27.7)	32 (32.3)	2.0 (1.0-4.2)	2.2 (1.1-4.5)*
≥3				20 (9.1)	18 (18.2)	3.5 (1.5-8.3)*	3.6 (1.5-8.7)*
<1				54 (24.6)	14 (14.1)	1.0	1.0
≥1				166 (75.4)	85 (85.9)	2.0 (1.0-3.8)*	2.1 (1.1-4.0)*

Abbreviation: OR, odds ratio; CI, confidence interval; AOR, adjusted odds ratio.

* *p* < 0.05.

1 McNemar's test: Tumor parts *vs.* their adjacent normal parts, *p* value < 0.0001 (n = 126; 84- and 42-paired samples from USA and Taiwan respectively).

2 Adjusting for age and sex

3 Case 2 *vs.* Control.

### Additional validation of ATP1A1 by array information from 53 Chinese ESCC patients reported by Su *et al* [18]

In the 53 Chinese ESCC patients (58% male; average age 58 yrs) studied by Su et al [18], the mean and median ATP1A1 tumor/normal ratios were 1.57 and 1.43 respectively, both significantly different from 1.00 (p < 0.0001) (Supplementary Figure S3).

### Serum ATP1A1 levels and the survival of ESCC patients

**Figure 3:**
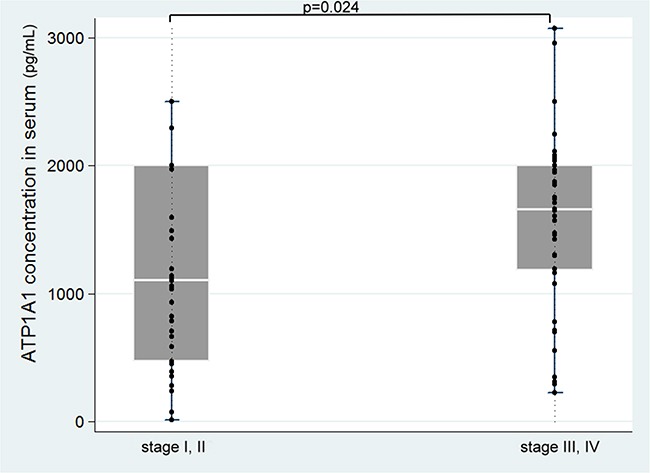
Serum ATP1A1 levels in different clinical stages of the disease

In the serum specimens we collected from 78 ESCC patients, we found that mean and median ATP1A1 levels to be 1,362.9 and 1,456.0 pg/mL (range 13.0 to 3,075.3 pg/mL). Patients with stage III/IV diseases had significantly higher ATP1A1 levels that those with stage I/II diseases (Figure 3). Mean ATP1A1 levels were not associated with age, sex, smoking, alcohol use, or betel nut use (Supplementary Table S5). Using the median level of 1,456.0 pg/mL as the cut-off point, we found that patients with stage III-IV were 2.9-fold more likely (95% CI=1.1-7.4, p=0.025) to have high serum ATP1A1 levels than those with stages I-II, after adjusting for age and sex. The results remained similar after further adjusting for other covariates. Kaplan–Meier plot dichotomized by median level of ATP1A1 (1,456.0 pg/mL) did not show the survival periods to be significantly different (Supplementary Figure S4). The Cox regression model showed clinical staging, but not ATP1A1 levels, to be predictive of survival (Supplementary Table S6).

### *In-vitro* studies

#### ATP1A1 expression in one ESCC cell line and its subpopulation

**Figure 4:**
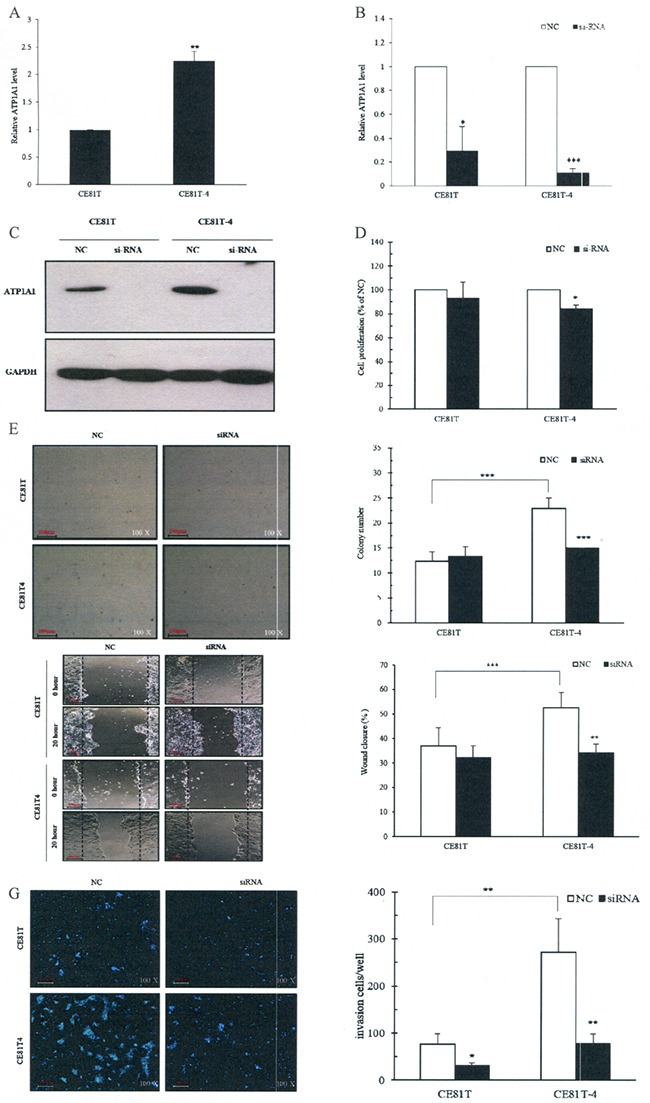
Downregulation of ATP1A1 expression by small interfering RNA (siRNA) decreases ESCC cell invasion, migration and colony formation *in vitro* **A.** Endogenous ATP1A1 RNA expression in the CE81T and CE81T-4 cells; **B.** Down-regulated ATP1A1 mRNA levels by ATP1A1 siRNA; **C.** Down-regulated ATP1A1 protein expression by ATP1A1 siRNA; **D.** Inhibition of cell proliferation in CE81T and CE81T-4 cells by ATP1A1 siRNA; **E.** Decreased colony formation in CE81T and CE81T-4 cells by ATP1A1 siRNA. **F.** Decreased cell migration ability using wound-healing assay in CE81T and CE81T-4 cells by ATP1A1 siRNA (Original magnification: ×200). **G.** Decreased invasion ability in CE81T and CE81T-4 cells by ATP1A1 siRNA. Data are quantified using Image J software and error bars represent s.d. of means for experiments in triplicate (***P value<0.005,**P value<0.001, *P value<0.05, respectively, Student's t-test).

Quantitative real-time PCR studies found that the expression of ATP1A1 RNA in CE81T-4 cells was significantly upregulated compared to CE81T (2.25 ± 0.17 *vs* 1.0 ± 0.00, Figure 4A). The CE81T-4 cells were more invasive than the parenteral CE81T cells [19].

### ATP1A1 siRNA silences ATP1A1 function resulting in ESCC cell proliferation, colony formation, migration, and invasion

After ATP1A1 siRNA was transfected into CE81T and CE81T-4 cells, we found that ATP1A1 RNA levels decreased to a ratio of 0.3 ± 0.2 in CE81T cells and 0.11 ± 0.04 in CE81T-4 cells, compared to negative controls (NC) (Figure 4B). Similar findings were also found in ATP1A1 protein expression (Figure 4C).

Observations using crystal violet found that downregulation of ATP1A1 by siRNA significantly reduced the proliferation rate in CE81T-4 cells, but not CE81T cells, compared with NC siRNA transfected cells at 48 hours (Figure 4D). Figure 4E shows the colony formation capacity significantly increased in CE81T-4 cells, when compared to CE81T cells, at day 14 and down-regulation of ATP1A1 in CE81T-4 cells by ATP1A1 siRNA resulted in a clear reduction of colony formation capacity, suggesting that ATP1A1 expression influences the growth and proliferation of CE81T-4 cells (Figure 4C and 4D).

 Figure 4F and 4G, which depicts the results of our wound-healing and Matrigel-coated Transwell member assays, show that CE81T-4 was significantly more cable to migrate and invade than CE81T. Downregulation of ATP1A1 by ATP1A1 siRNA significantly inhibited cell migration and invasion ability in CE81T-4 cells, although its inhibiting effects were not as strong in CE81T.

### Ouabain suppresses ESCC cell proliferation, colony formation, migration and invasion

**Figure 5:**
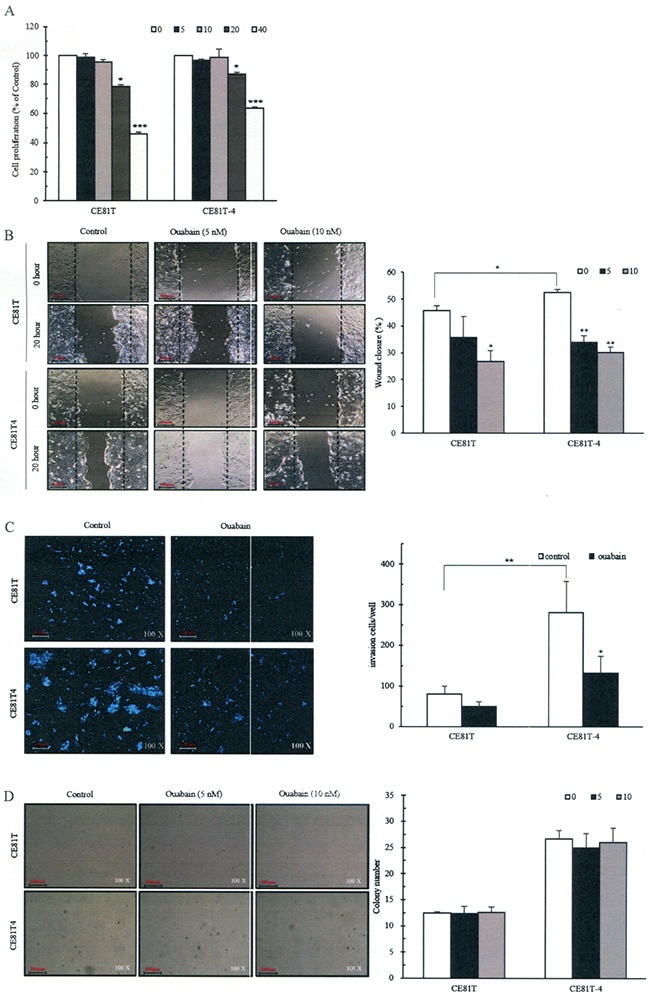
Inhibitions of ESCC cell proliferation, migration and invasion and colony formation by ouabain **A.** Different concentrations of ouabain on cell proliferation; **B.** Confluent monolayers of CE81T and CE81T-4 cells were wounded using a 1-mm-wide tip and incubated with ouabain (0–10 nM) for 16 hours. The wound space was analyzed and represented as the migration level relative to the change of the untreated cells; **C.** Ouabain (0-5nM) reduced cell invasion of the CE81T and CE81T-4 cells as revealed by a trans-well invasion assay; **D.** Effects of ouabain on colony formation. Cells were seeded in a six-well plate culture (2× 10^5^ cells/well) and incubated for 10 days to allow colony formation. Migratory or invasive cells were imaged in a bright-field microscope under × 20 magnification. Quantitative determination of colony numbers in ouabain treatment cells. Cell number (≥50 μm) in a cluster was defined as a signal colony. Data were quantified using ImageJ software and error bars represent s.d. of means for experiments in triplicate (***P value<0.005,**P value<0.001, *P value<0.05, respectively, Student's t-test).

To examine cytotoxicity of ouabain, we used four different biological concentrations of ouabain (5, 10, 20 or 40 nM) to treat CE81T and CE81T-4 cells for 48 hours. We observed their growth rate and found that ouabain decreased cell growth rate in a concentration-dependent manner (-1.32 ± 2.37%, -4.61 ± 2.29%, -21.6 ± 7.79%, and -54.15 ± 2.42% in CE81T cells and -3.26 ± 1.89%, -1.21 ± 2.93%, -12.86 ± 2.72%, and -36.50 ± 1.55% in CE81T-4 cells, respectively, Figure 5A). To avoid the cytotoxicity of high-concentration ouabain in ESCC cells, the subsequent experiments used 5 and 10 nM ouabain. Figure 5B shows 10 nM ouabain significantly inhibited cell migration in both CE81T and CE81T-4 cells with inhibition rates of 40.29 ± 9.00% and 43.76 ± 3.55%, respectively, although 5 nM ouabain only significantly inhibited migration rate in CE81T-4 cells (35.36 ± 4.45%). Similar results were also noted in Matrigel-coated Transwell member invasion assay (Figure 5C). In contrast, 5 nM and 10 nM ouabain did not substantially inhibit soft agar colony formation of CE81T and CE81T-4 cells (Figure 5D), although these two cell lines have distinct sensitivities to ouabain.

## DISCUSSION

By combining microarray-based screening of esophageal tumors from both NMBA- and arecoline-induced F344 rats and 17 human ESCC specimens, we identified four upregulation candidate genes related to secretory or membranous proteins. Of these genes, ATP1A1 was overexpressed in both human tissue and serum specimens supplied from donors from different countries, and this overexpression was associated with the development and progression of ESCC, although serum ATP1A1 expression did not predict survival. However, in our *in-vitro* study, we found that downregulation of ATP1A1 by ATP1A1 siRNA and the inhibitor ouabain could inhibit the migration and invasion ability of ESCC cell lines. Thus, ATP1A1 may potentially be used in the diagnosis and grading of ESCC. To best of our knowledge, this is the first study to examine the relationship between ATP1A1 expression and the risk and progression of ESCC.

NMBA, one of the N-nitrosamino acids in tobacco and/or tobacco smoke, is a powerful and specific carcinogen for esophagus and has been extensively used to study esophageal carcinogenesis in rats [20, 21]. Because previous human studies have found that cigarette smoking plus areca nut chewing increases and enhances the esophageal carcingoensis [2, 22, 23], we added arecoline, the major alkaloid found in the areca nut, in our NMBA-induced F344 model to mimic real situations in humans. We found that F344 rats treated with NMBA plus arecoline developed more esophageal tumors than those treated with NMBA only over a 30-week observational period and that all seven F344 rats treated with NMBA plus arecoline developed esophageal tumors, but not in the other groups over a 25-week observation period. Those results were consistent with our previous case-control studies in humans, suggesting arecoline may serve as a promoter accelerating or increasing the tumor formation of esophagus [2, 22, 23].

One previous study used another murine model to examine the combined effect of 4-nitroquinoline 1-oxide (4-NQO) and arecoline on oral carcinogenesis [6]. In that study, Chang and his colleagues used C57BL/6JNarl mice treated with arecoline, 4-NQO, or both in high and low doses for 8 weeks to observe the induction of oral tumors over an additional 20-week follow-up period. They reported a 100-percent incidence rate for tumor in the tongue of mice mice exposed to both 4-NQO (200 μg/ml) and arecoline (500 μg/mL), 57% in mice exposed to 4-NQO only, and 0% in mice exposed to arecoline only. However, they did not find esophageal tumor in any of the experimental groups, including the group of mice exposed to both 4-NQO and arecoline, suggesting this mouse model is not ideal for the study of esophageal tumorigenesis [6]. Thus, the murine model we established in this investigation has become an important *in-vivo* tool to study esophageal carcinogeneis and its mechanism induced by both NMBA and arecoline.

ATP1A1 is one of three subunits of Na^+^/K^+^-ATPase. In addition to its classical functions such as ion transport, generation of a membrane potential and nutrient uptake, Na^+^/K^+^-ATPase also plays roles in signal transduction, cell junction, cell adhesion and migration [8]. ATP1A1 consists of two essential non-covalently bound subunits, a1 and b1. ATP1A1 is the catalytic subunit, and ATP1A1 b1 is involved in the translation, stability, and transport of a1 subunit on the plasma membrane [11]. Aberrant expressions of both ATP1A1 a1 and ATP1A1 b1 have been reported in various cancers [8]. One recent study showed proliferation arrest and reduced cell migration of human HCC cells after knockdown of ATP1A1 expression [24]. The tumorigenicity of the HCC cells in nude mice was reported to be markedly decreased (100% *vs.* 10%) after being transfected with ATP1A1-shRNA [24]. Moreover, adding specific inhibitors of Na^+^/K^+^-ATPase (e.g., ouabain or digoxin) to certain cells can activate multiple signaling pathways, including the EGFR/Src-Ras-Erk pathway and the PI3K-PDK-Akt pathway [25, 26]. Thus, the known mechanisms through which ATP1A1 probably affects tumorigenicity and metastasis include (1) affecting K^+^ homeostasis resulting in cell apoptosis or necrosis [25, 26], (2) affecting the translocation of fibroblast growth factor 2 (FGF2) from cytoplasm to extracellular and PI3K/Akt signaling pathway to influence cell proliferation, migration, and invasion [27], and (3) interacting with ATP1A1 α1-subunit and phosphoinositide 3-kinase (PI3K) regulatory unit to control focal adhesion kinase (FAK) phosphorylation in lamellipodia or invadopodia [10].

Consistent with previous studies of other cancer cell lines, our *in-vitro* experiments showed that ATP1A1 expression is highly correlated with the invasive ability of a subpopulation from the same ESCC cell line (CE81T *vs.* CE81T-4) established in our laboratory [19]. By knocking down ATP1A1 with siRNA or ouabain, we were able to significantly reduce CE81T-4's ability to migrate and invade, compared to controls. The results of our cell proliferation assay showed that ATP1A1 siRNA, but not ouabain, effectively knocked down cell proliferation rate in CE81T-4. One possible explanation for this discrepancy in findings may be related to the fact that ouabain induces certain intrinsic signaling pathways, such as Src-Ras-Erk and PI3K-PDK-AKT pathways [10]. These pathways are known to enhance cell survival and proliferation and thus may be able to counteract the negative effects of Na^+^/K^+^ homeostasis disruption.

This study has some limitations. One is that the human studies were cross-sectional, and as such they cannot be used to definitely conclude a causal link between ATP1A1 expression and the risk and progression of esophageal neoplasm. We found an association between serum ATP1A1 expression and clinical staging, but not survival. This discrepancy may be due to small sample size or other unknown factors. Because we only measured serum ATP1A1 expression in ESCC patients, whether the presence of ATP1A1 upregulation in patients with Barrett's esophagus is unknown. Thus, we cannot determine whether upregulation of ATP1A1 in serum is ESCC-specific. Still another limitation is that only one measurement of ATP1A1 expression in serum was available in this study. The future studies should collect a series of measurements of ATP1A1 in patients before and after cancer treatment to further confirm the role of ATP1A1 as a tumor marker.

In conclusion, our findings suggest that ATP1A1 expression might potentially be helpful in the diagnosis and staging of esophageal cancer. Prospective studies could be designed to confirm whether ATP1A1 expression can predict the risk and progression of ESCC in high-risk populations. A more detailed study of its mechanism is also needed.

## MATERIALS AND METHODS

### Animals

Four-week-old male F344 rats were purchased from Taiwan's National Laboratory Animal Center (Taipei, Taiwan) and were handled in accordance with the Animal Care and Use Guidelines of the Kaohsiung Medical University. Rats were kept under standard conditions (20 ± 2°C, 50 ± 10% relative humidity, 12 hour light/dark cycle) and given standard rat chow (Altromin'de Industry Co. Ltd., Munich, Germany) and water *ad libitum*. They were housed in solid-bottomed polycarbonate cages in groups of two or three. Body weight and water intake were recorded weekly. Chemicals used in this study were described in the Supplementary Appendix.

### Experimental protocol

We used N-benzyl-N-methylnitrosamine (NMBA)-induced F344 rats with or without co-treatment of arecoline, a major ingredient of areca nut, to observe the development of esophageal papilloma over 25 or 30 weeks (Supplementary Figure S5). One previous study showed that a 0.5 mg/kg dosage of NMBA subcutaneously injected (sc) three times a week for five weeks in F344 rats can induce esophageal tumors within 30 weeks in all their rats [28]. The current study followed a similar protocol. Specifically, 88 four-week-old rats were stabled and given drinking water and standard rat chow for two weeks before the experiment (Supplementary Figure S6). Then the six-week-old rats were randomly assigned into four groups-Control, NMBA, Arecoline, and NMBA+Acrecoline Groups. The control group (n = 22) received 0.2 mL of a solution containing 20% DMSO in water (the vehicle for NMBA) by sc three times a week in the first five weeks and freely accessible arecoline-free drinking water for the entire experimental period. The NMBA group (n = 22) received 0.5 mg/kg concentrations of NMBA by sc three times a week in the first five weeks and freely accessible arecoline-free drinking water for the entire experimental period. The Arecoline group (n = 22) received 0.2 mL of a solution containing 20% DMSO in water by sc three times a week in the first five weeks and freely accessible 500 μg/mL arecoline drinking water for the entire experimental period. The NMBA + Arecoline group (n = 22) received 0.5 mg/kg concentrations of NMBA by sc three times a week in the first five weeks and freely accessible 500 μg/mL arecoline drinking water for the entire experimental period. The drinking water with or without arecoline was changed three times a week. Seven rats from each group were sacrificed at the end of the 25th week, while the remaining 15 rats in each group were observed for an additional five weeks and then sacrificed at the end of the 30th week in order to examine tumors of different organs, including esophagus [29].

All rats were killed by CO_2_ asphyxiation. The esophagus of each animal was removed and washed with phosphate-buffered saline (PBS), opened longitudinally, and fixed using a needle on a sheet of cork oak. The surface of papillomas were mapped and counted. According to a previous study [29], lesions ≥ 0.5 mm in single dimension (length, width, and height) were considered to be papilloma. One half of the identified esophageal papilloma tumors and their normal esophageal parts were randomly selected from the rats of each group, pooled, and prepared for subsequent microarray analysis. The other half were fixed in 10% formalin and processed for histopathological examination.

A macroscopic inspection of the tongue, stomach, liver and lung was also performed. In addition to esophagus, we also fixed the tongue, stomach, liver and lung in 10% formalin and processed them for histopathological examination. For histopathological examinations, each organ we studied was cut into pieces and transferred to a cassette. The cassette was immersed in baths of ethanol to dehydrate the tissue followed by the addition of toluene. The paraffin-embedded sample tissue was sliced by microtome to layer on a glass for staining. Finally, esophageal specimens were stained with hematoxylin and eosin (HE) for histopathological examination. This histopathological examination was performed by two pathologists (CC Wu and CY Chai) who were blinded to the experimental groups.

### Oligonucleotide DNA microarrays for esophageal tissues of rats

The Rat Whole Genome OneArray® v1 (Phalanx Biotech Group, Taiwan) contains 25,338 DNA oligonucleotide probes. Among the probes, 24,358 probes correspond to the annotated genes in RefSeq v42 and Ensembl v59 database. Another 980 control probes are also included. Detailed descriptions of the gene array list can be viewed at http://www.phalanxbiotech.com/products/ROA.php.

Fluorescent aRNA targets were prepared from 1 μg total RNA samples using OneArray® Amino Allyl aRNA Amplification Kits (Phalanx Biotech Group, Taiwan) and Cy5 dyes (Amersham Pharmacia, Piscataway, NJ, USA). Fluorescent targets were hybridized to the Rat Whole Genome OneArray® with Phalanx hybridization buffer using the Phalanx Hybridization System. After 16 hours hybridization at 50°C, non-specific binding targets were washed away in three different washing steps (wash at 42°C for 5 minutes, wash at 25°C for 5 minutes, and rinse 20 times), and the slides were dried by centrifugation and scanned by an Axon 4000B scanner (Molecular Devices, Sunnyvale, CA, USA). The Cy5 fluorescent intensities of each spot were analyzed by GenePix 4.1 software (Molecular Devices, Sunnyvale, CA, USA). The signal intensity of each spot was loaded into Rosetta Resolver System® (Rosetta Biosoftware, Seattle, WA, USA) to perform data analysis. The error model of Rosetta Resolver System® can remove both systematic and random errors from the data. Spots where the flags were less than 0 were filtered out. Spots that passed the criteria were normalized by the 50% media scaling normalization method. The technical repeat data was tested by Pearson correlation coefficient calculation to check the reproducibility (R value > 0.975). Normalized spot intensities were transformed to gene expression log2 ratios between the control and treatment groups. The spots with log2 ratios ≥ 1 or log2 ratios ≤ ‐1 and P-values < 0.05 were further analyzed.

### Oligonucleotide DNA microarrays for 17-paired human ESCC tumor and normal tissues

The tumor tissues and distant normal tissues from 17 ESCC patients from Kaohsiung Medical University Hospital (KMUH) before any cancer treatment were assayed by Human oligonucleotide DNA microarrays (Human Whole Genome OneArray^TM^) from Phalanx Biotech Group (Hsinchu, Taiwan). The procedures have been described previously [30]. The raw data has been uploaded to the Gene Expression Omnibus (GEO) database [31]. In brief, the Human Whole Genome OneArrayTM contained 32,050 60-mer oligonucleotide probes with 28,703 probes corresponding to the annotated genes in Unigene v175 and RefSeq database. One-half μg RNA of each sample was amplified by Illumina TotalPrep RNA Amplification Kit according to manufacturer's instructions (Ambion, Austin, TX, USA). Then, 10 μg of fragmented biotin-labeled cRNA was hybridized on Phalanx Human OneArray™ by Phalanx hybridization buffer at 50°C in an oven for 14-16 hours using the bubble-mixing method. After non-specific binding targets were washed, the hybridization arrays were conjugated with fluorescent detector of Strepavidin-Cy3. Finally, arrays were dried by centrifugation and scanned by DNA Microarray Scanner (Agilent Technologies, Santa Clara, CA, USA). Images from the scanned arrays were quantified using GenePix^®^ Pro 4.1.

### RNA extraction, reverse transcription, and quantitative PCR (qPCR) in esophageal tumor and adjacent normal tissues

The extraction of tissue RNA followed the instruction of manufactures' protocol (RNAeasy mini kit, code:74104, QIAGEN). Tissue specimens were cut into slices less than 0.5 cm thick, separately pooled, and soaked with RNAlater (RNA Stabilization Reagent, cat:76104, QIAGEN) which approximately contained 10 μL reagent per 1 mg tissue. Before starting homogenization, we removed RNAlater reagent and determined the amount of tissue under 30 mg; then, we placed the weighted sample into vessel, mixed with 600 μL RLT buffer (RNAeasy mini kit, code:74104, QIAGEN) containing 0.1% b-mercaptoethanol, homogenized the tissue with Precellys 24 tissue homogenizer (Bertin Technologies, French) under 6000 g for three times, and centrifuged the lysate and transferred clear supernatant into a new microtube. Finally, the concentration of RNA was measured by a NanoDrop Spectrophotometer ND-1000 (Thermo Fisher Scientific Inc.).

All of the reverse transcription reactions were performed by Reverse Transcription System (Cat. no: A3500, Promega, Madison, WI, USA). In order to achieve optimal reverse transcription efficiency, 1 μg total RNA was placed in a microcentrifuge tube and incubated at 70°C for 10 min in a thermocycler (Gene Amp PCR system 9700, Applied Biosystems, Foster City, CA, USA). After denaturing the secondary structure of RNA, the thermocycler was rapidly set at 4°C to wait for the next step. As described in manufacturer's instructions, the final concentration of reaction component was 5 mM MgCl_2_, 1× Reverse Transcription Buffer (10 mM Tris-HCl [pH 9.0 at 25°C], 50 mM KCl, 0.1% Triton® X-100), 1 mM each dNTP, 1 u/μL Recombinant RNasin® Ribonuclease Inhibitor, 15 u/μg AMV Reverse Transcriptase (High concentration), and 0.5 μg Oligo (dT)_15_ primer. The reaction mixture was loaded into a microcentrifuge tube mixed with total RNA, incubated at 42°C for 15 min, heated to 95°C for 5 min to inactivate AMV reverse transcriptase, and finally stored the first-strand cDNA at -20°C until use.

ATP1A1 expression was quantified with TaqMan gene expression assay reagents (*ATP1A1* gene expression ID: Hs0016755_m1; Applied Biosystems, Foster City, CA, USA) according to the instruction of manufacturer. In brief, the reaction mix included 100 ng cDNA sample, the TagMan Universal Master Mix and 20× assay to the volume of 20 μL for real-time PCR on the system of StepOne™ Real-Time PCR (Applied Biosystems, Foster City, CA, USA). Each sample was run duplicatedly. The PCR reaction cycles were 40 under the condition of denaturion at 95°C for 15 seconds; annealing and extension at 60°C for 1 minute; and the data was analyzed by StepOne™ v2.3 software (Applied Biosystems, Foster City, CA, USA).

### Additional validation of ATP1A1 expression by array information of 53 Chinese ESCC patients [18]

The global gene expression profile of 53 ESCC samples and 53 matched normal samples was retrieved from http://www.ncbi.nlm.nih.gov/geo/query/acc.cgi?acc=GSE23400 to examine tumor-to-normal ratio of ATP1A1 expression. These 53-pair samples were analyzed using an Affymetrix GeneChip Human Genome U133 Set (HG_U133A and HG_U133B, Affymetrix).

### Immunohistochemistry (IHC) staining of tissue array for ATP1A1 in esophageal tissue

IHC was used to assess ATP1A1 protein in ESCC tissue arrays from several different sources, KMUH (n = 19) in Taiwan, China Medical University Hospital (CMUH, Taichung, n = 29) in Taiwan, and two commercial companies from Korea and USA (Supplementary Table S3).

All study subjects from Taiwan provided written informed consent. Because the patient identifiers of the commercial tissue arrays were scrambled to the public for research purposes, no written informed consent was required. The protocol of this study was approved by the Institutional Review Board of Kaohsiung Medical University Hospital.

The tissue arrays from KMUH and CMUH were collected 2005-2008. They consisted of 19 and 29 resectable ESCC specimens along with their distant normal pairs formalin-fixed and paraffin-embedded (FFPE). The clinicopathologic characteristics of the 48 ESCC patients, different from those collected of the 53 Chinese patients mentioned above [19], are shown in Supplementary Table S7. MTA Booster and the software of TMADesigner software were used to arrange the tissues for the array. We first used the puncher to punch the selected blocks, which had been labeled the tumor sites and non-tumor sites (distant normal ones), to obtain the tissue cylinders (3 mm in height and 2 mm in diameter) and arranged them on the slide in order. Each slide contained two cancer tissues and two distant normal tissues in the same subject from 15 ESCC patients (Supplementary Figure S7).

Another two sets of esophageal cancer tissue array specimens were purchased from two companies: SuperBioChips Laboratories (Seoul, Korea) and US Biomax, Inc. (Rockville, MD, USA). The CR2 specimens from SuperBioChips Laboratories consisted of 60 cores (solely tumor specimens) from 60 individual ESCC patients. Each core was 2 mm in diameter and 4 μm in thickness. From US Biomax, we obtained five different tissue array slides, including ES801 (80 cores of paired tumor and normal parts from 40 ESCC patients), ES802 (80 cores with duplicated tumor specimens without normal parts from 40 ESCC patients), ES1501 (150 cores with duplicated tumor specimens without normal parts from 75 ESCC patients), ES2001 (200 cores from 120 ESCC patients), and BC02022 (54 cores containing tumor specimen, adjacent normal, and distant normal parts from 18 ESCC patients) (Supplementary Table S1).

We first deparaffinized the specimens through serial baths in xylene and rehydrated them in a graded series of baths in alcohol and water. The tissue block was then autoclaved in target retrieval solution (Target Retrieval Solution pH 9, Code: S2368, Dako, Glostrup, Denmark) at 121°C and 15 psi for 20 min to improve the efficiency of antigen-antibody reaction. To remove endogenous peroxidase activity and nonspecific background staining, the specimens were treated with 3% hydrogen peroxide for 5 minutes at room temperature. After three times TBS washing, Na+/K+-ATPase α (sc-28800, SantaCruz, CA; dilution at 1: 300) was mounted and incubated on a slide with coverslip at room temperature. One hour later, the section was rinsed twice with TBS, and anti-mouse/rabbit secondary antibody conjugated with HRP (ChemMate™ DAKO EnVision™ Detection Kit, Code: K5007, DAKO) was added for 30 minutes incubation. Finally, the specimen was washed twice with TBS and visualized by incubating with 0.03% 3,3’-diaminobenzidine tetrahydrochloride (ChemMate™ DAKO EnVision™ Detection Kit, Code:K5007, Dako) for 5 minutes. All slides were lightly counterstained with Mayer's hematoxylin for 30 seconds, washed in running water, dehydrated, and mounted with Canadian balsam.

The IHC ATP1A1 staining scores in high-power field (200×) areas of specimens of each case patient were assigned by two independent pathologists (CC Wu and CY Chai) blinded to the experimental groups and patients' clinical status. If the readings were not consistent, findings were discussed until final score was made by consensus. ATP1A1 intensity score was calculated by averaging cytoplasmic and membrane staining of positive cells in cancer and normal tissues separately as follows: 0 = negative staining, 1 = focal weak cytoplasmic staining, 2 = diffuse moderate cytoplasmic staining and blurred membrane staining, and 3 = diffuse strong cytoplasmic stain and distinct membrane staining (Supplementary Figure S8) [32]. For those subjects with two different IHC scores for two different specimens, mean IHC score was used.

### Serum ATP1A1 measurement by ELISA

Serum specimens from ESCC patients were stored in a freezer at -80°C until analysis. ATP1A1 protein levels were measured by the ELISA to quantify human sodium/potassium-transporting ATPase subunit alpha-1 concentrations (CUSABIO^®^, Catalog Number: CSB-EL002322HU, Wuhan, Hubei Province 430223, P.R.China). The detection limits of serum ATP1A1 were 16.5 pg/mL according to manufacturer's directions. The coefficients of variations [CV (%)] of intra-assay and inter-assay precisions were < 8% and < 10%, respectively. One technician (HS Lin), who was blinded to the clinical staging of ESCC patients, performed the measurements.

### *In-vitro* studies

#### Cell culture and cell proliferation

Human esophageal squamous cell carcinoma cells (CE81T/VGH and CE81T-4 subline) were obtained from the Bioresource Collection and Research Center (BCRC, Hsinchu, Taiwan), and cultured in Dulbecco's Modified Eagle Medium (DMEM, GIBCO, Grand Island, NY, USA) supplemented with 10% fetal bovine serum (FBS), 1% MEM non-essential amino acids (NEAA) and 1% antibiotic-antimycotic and then kept at 37°C in a humidified atmosphere with 5% CO_2_incubator to maintain exponential growth.

Cells were seeded in 24 well plates (5× 10^4^cells/well) and they were treated with different concentrations of ouabain octahydrate (ATP1A1 inhibitor) as described previously [33] or ATP1A1 siRNA for 24, 48 and 72 hours (Supplementary Appendix) Cells were fixed in 4% paraformaldehyde and stained with 0.1% crystal violet for 15 min at room temperature. The intensity of crystal violet was quantified by the absorbance of 590 nm after dissolving the stained cells with 0.1% Triton X-100. Details about transfection and migration and invasion assays are described in the Supplementary Appendix.

### Statistical analysis

In our animal experiment, we wanted to test whether the NMBA + Arecoline group would develop more tumors or develop tumors earlier than the NMBA group. We also analyzed the differences in tumor development across the four groups. First, body weights and papilloma multiplicity were expressed as mean ± standard deviations (SD). The average number of esophageal papilloma between groups was examined by one-way ANOVA, and significance was assessed by the LSD post hoc test. Group differences in incidence of papilloma were analyzed by χ^2^ statistics.

In our study of the human specimens, we categorized IHC scores into grades of < 1, 1-< 2, 2-< 3, and ≥ 3 first in normal and tumor tissues separately. Because all the IHC scores of ATP1A1 in normal tissues were graded as 0 or 1, we also categorized all normal and tumor parts by ≥1 *vs.* < 1 first. McNemar's test was performed to determine the difference between tumor *vs.* normal tissues (distant or adjacent normal tissues) in the matched pairs, whereas χ^2^ test was used to compare the differences between case patients and controls. Multivariate logistic regression was used to investigate the relationship between IHC score and the development of ESCC after adjusting for other covariates, including age, gender, clinical staging, and source of specimens. We also examined the relationship between IHC intensity score and clinical staging (stages I and II *vs.* stages III and IV) after adjusting for age, gender, and source of specimens.

Serum ATP1A1 levels were dichotomized based on their mean and median values. Using median ATP1A1 level as the cut-off point, we examined its relationship with clinical staging (stages I/II *vs.* III/IV) before and after adjusting for age and sex. To examine whether median serum ATP1A1 levels predicted the survival of ESCC patients, we used Kaplan–Meier analysis and log-rank testing in crude analysis and Cox proportional hazards modeling with computing hazard ratios (HRs) and 95 % CIs in multivariable analysis. Each participant accumulated person-time beginning from the date of ESCC diagnosis and ending on the date of ESCC death or the end of this study in January 2016. We excluded the ESCC patients who died within one month (N=3) or who were lost to follow-up (N=1) after initial cancer diagnosis because they usually had very poor performance, severe infection, or refused cancer treatment. The covariates in Cox regression included the above-mentioned variables in logistic regression plus clinical staging. All statistical operations were performed using the SAS (Version 9.3) statistical software package; a *p-*value of less than 0.05 was considered significant.

## SUPPLEMENTARY MATERIALS APPENDIX






