# Supplementary material for: Overexpression of ATPase Na+/K+ transporting alpha 1 polypeptide, ATP1A1, correlates with clinical diagnosis and progression of esophageal squamous cell carcinoma
Source: Oncotarget. 2016 Nov 10;7(51):85244–58. doi: 10.18632/oncotarget.13267 (PMC5356733; doi:10.18632/oncotarget.13267)
Supplement: Supplementary file 1 [file oncotarget-07-85244-s001.pdf]

Overexpression of ATPase Na+/K+ transporting alpha 1 polypeptide, ATP1A1, correlates with clinical diagnosis and progression of esophageal squamous cell carcinoma

SUPPLEMENTARY APPENDIX

MATERIALS AND METHODS

Chemicals

Arecoline and dimethylsulfoxide (DMSO) were purchased from Sigma-Aldrich (St. Louis, MO, USA) and N-benzyl-N-methylnitrosamine (NMBA) was obtained from Nard Chemical Co. Ltd. (Osaka, Japan).

RNA preparation for esophageal tumor and normal tissues

After excising tumor or normal tissue samples from esophagus, these two tissue specimens were rapidly cut into slices less than 0.5 cm thick, separately pooled, and soaked with RNAlater (RNA Stabilization Reagent, cat:76104, QIAGEN) which approximately contained 10 µL reagent per 1 mg tissue. Before starting homogenization, we removed RNAlater reagent and determined the amount of tissue under 30 mg; we placed the weighted sample into vessel and mixed with 600 µL RLT buffer (RNAeasy mini kit, code:74104, QIAGEN) containing 0.1% β-mercaptoethanol. Then, we disrupted and homogenized the tissue with Precellys 24 tissue homogenizer (Bertin Technologies, French) under 6000 g for three times; centrifuged the lysate and transferred clear supernatant into a new microtube, mixing with 1 volume of 70% ethanol; then transferred 700 µL of sample into a spin column (RNAeasy mini kit, code:74104, QIAGEN) and centrifuged for 15 s at 8000 g. After three-step washing procedures instructed by the manufacturer, the spin column was placed into a new collection tube and added another 50 uL RNase-free water, centrifuged at 8000 g for 1 minute to elute RNA. The RNA concentration and purity were checked by an Agilent 2100 Bioanalyzer (Agilent Technologies, Santa Clara, CA, USA), which OD260/OD280 and (> 1.8) and OD260/OD230 should meet > 1.8 and > 1.6, respectively, for the subsequent array experiment.

In-vitro studies

Transfection of ATP1A1 siRNA (gene silencing)

Cells were seeded in 6 well plates (5×10^5 cells/well) and incubated with the reagent of transfection complexes with five nM ATP1A1 small interfering RNA (siRNA; oligo ID s1718; 4390824, Ambion/life technologies, Carlsbad, CA, USA) and 12 µl HiPerFect to serum-free medium. After 24 hours, cells were processed. Transient transfection of scramble siRNA and ATP1A1 siRNA molecules was carried out using HiPerFect reagent (Qiagen, Valencia, CA) following the procedure provided by the manufacturer.

Na^+/K^+-ATPase inhibitor-ouabain

We determined the IC_{50} of ouabain by 2-fold serial dilution on 96-well plate for 48 hours. First, the frozen reagent of CellTiter 96® Aqueous One Solution was thawed at 37°C water bath for 10 minutes; then 20 µL reagent was pipette into each well with multichannel pipette; the 96 well plate was incubated at 37°C for 2 hours in a humidified with 5% CO_2; and the absorbance at 490 nm was recorded using a 96-well plate reader. The IC_{50} was determined by standard curve with $P > 0.001$. We chose 5 nM and 10 nM ouabain for the subsequent invasion, wound healing, and colony formation assays due to their low cell toxicity.

Cell migration assay

Cell migration assay was conducted using the trans-well chamber inserts (8 µm pore size; PIEP12R48, Millipore, Billerica, MA, USA). siRNA transfected cells were resuspended in 300 µL serum-free medium, added to the upper chamber (1×10^5 cells/well) chamber with DMEM/10% FBS in the lower chamber. Following 16 hours incubation, cells on the lower membrane side were fixed in 4% paraformaldehyde and stained with the fluorogenic compound DAPI (4',6-diamidino-2-phenylindole). Then, the number of migratory cells in the lower chamber was counted using a microscope.

Wound-healing assay

Cells were seeded in 24 well plates with cell density of 5×10^5 cells/well for CE81T and 2×10^5 cells/well for CE81T-4. Once the cells reached 90% confluence, a scratch was carefully made using sterile 200-µl pipette tip to produce a linear wound area in the monolayer and then incubated for 20 hours. Cell migration toward the wounded area was observed and photographed. The healing process was monitored using the area measurements.

Trans-well invasion assay

Trans-well invasion assays were conducted using Corning® BioCoat™ Tumor Invasion (354166, Tewksbury, NY, USA) containing 8 µm pores. Except the chamber was additionally coated with matrigel, the procedure of invasion assay was similar to that of the migration assay.

Colony formation assay

Cells were seeded in 6 well plates (2× 10^5 cells/well) with a bottom layer of 1% bacto agar in DMEM and a top layer of 0.7% bacto agar in DMEM. Fresh DMEM with 10% FBS was added to the top layer of the soft agar. After 10 days, the cells were washed twice with PBS and fixed in 4% paraformaldehyde. The number of colonies (≥50 µm) was counted under a microscope. All these experiments were performed in triplicates.

Immunoblot analysis

The protein expression was detected by immunoblot analysis. The cell lysates with equal protein content were prepared in SDS sample buffer, separated on native PAGE novexw bis–tris 4–16% gel for BN-PAGE analysis (Invitrogen, Carlsbad, CA, USA) followed by transferring to polyvinylidene fluoride membrane. Proteins on the membrane were detected with specific primary antibodies and HRP-conjugated secondary antibodies. Signal of target protein was visualized by incubation with ECL Reagent and exposure to X-ray film.

RESULTS

Animal model

Body weight and water consumption

The mean body weight in the four groups followed an increasing trend with no loss till the end of termination at the 25th and 30th weeks (Supplementary Figure S6A), although rats in the groups of Arecoline and NMBA + Arecoline, on average, drank relatively lesser amounts of water than those in the other two groups (Supplementary Figure S6B).

Papilloma in the tongue and other organ sites

In the 25th week, we found that 1 (14.3%) and 3 (42.9%) out of all 7 rats in the NMBA and the NMBA + Arecoline groups respectively, developed tongue papilloma (Supplementary Table S1). In contrast, no esophageal papilloma was found in the control and the Arecoline groups. In the 30th week, 1 (6.7%), 5 (33.3%), and 10 (66.7%) out of 15 rats in the Arecoline, the NMBA, and the NMBA + Arecoline respectively, developed tongue papilloma, whereas no papilloma was found in the control group (Supplementary Table S3).

In the 25th week, the average numbers (± SE) of tongue papilloma per rat were 0 in the control and the Arecoline group, 0.29 ± 0.17 in the NMBA group, and 0.43 ± 0.17 in the NMBA + Arecoline group. In the 30th week, the average numbers (± SE) of tongue papilloma per rat were 0 in the control group, 0.07± 0.07 in the Arecoline group, 0.53 ± 0.22 in the NMBA group, increasing to 1.07 ± 0.30 in the NMBA + Arecoline group. The NMBA + Arecoline group had significantly more papillomas than in the NMBA group ($p = 0.0494$). In addition, The NMBA + Arecoline group had significantly more papillomas than the control group ($p = 0.0002$) and the Arecoline group ($p = 0.0004$). The NMBA group had more papillomas than the control group ($p = 0.0494$) and the Arecoline group, but this was not significant ($p = 0.0842$). The average number of tumors in the Arecoline group was similar to that in the control group ($p = 0.8026$) (Supplementary Figure S9). For other organ sites, including stomach, liver, kidney, and lung, no tumor was found in the 25th and 30th weeks.

SUPPLEMENTARY FIGURES AND TABLES

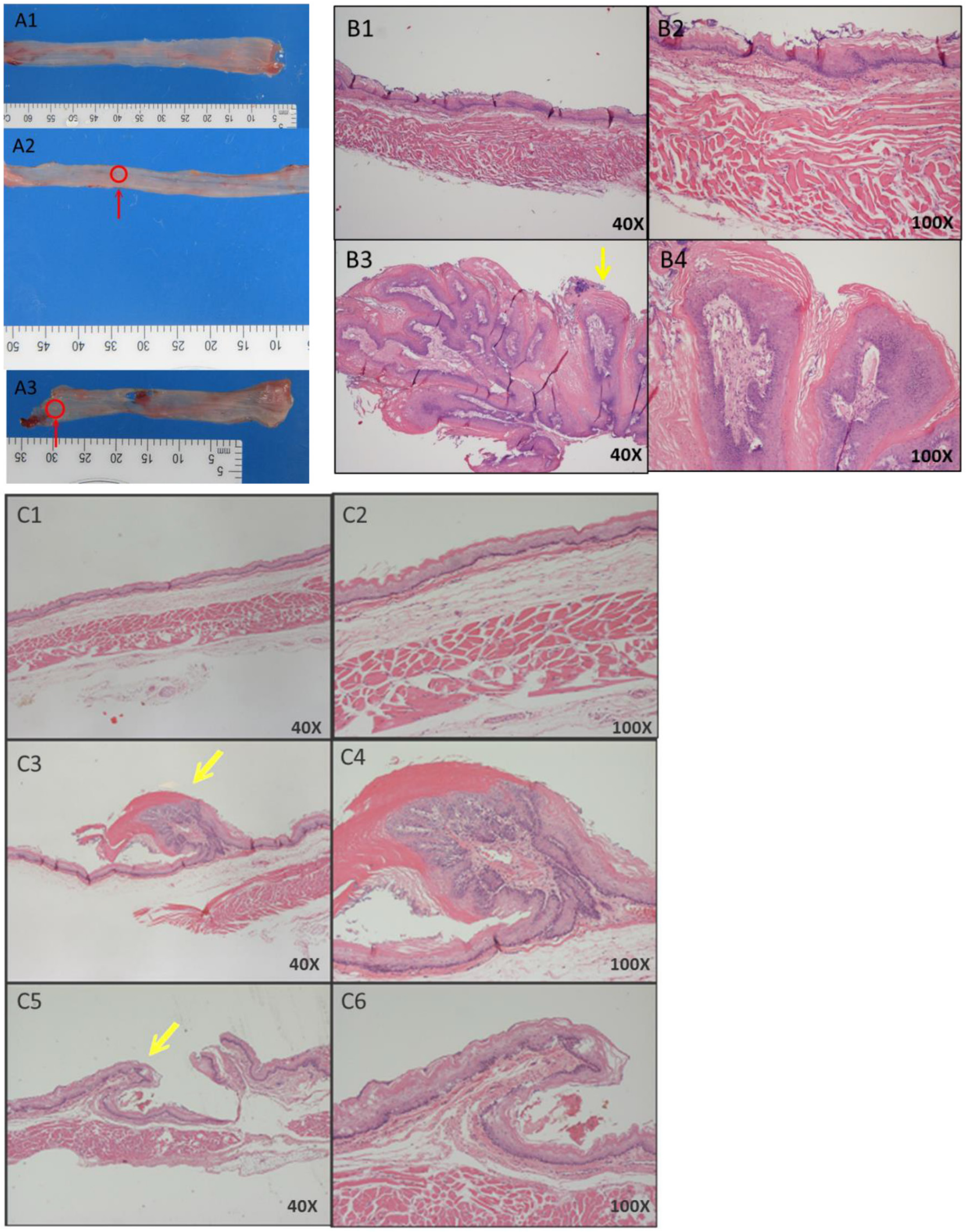

Supplementary Figure S1: Representative gross appearance and microscopically histological hematoxylin and eosin (HE)-staining in the resected esophageal tissues at the end of 25th and 30th weeks in different experimental groups. **A.** Papilloma and normal part of esophagus (arrow, papilloma). **A1.** One representative normal esophagus at the end of 25th week in the control group. **A2.** One representative esophageal papilloma at the end of 25th week in the NMBA + Arecoline group. **A3.** One representative esophageal papilloma at the end of 30th week in the NMBA + Arecoline group; **B.** Histological HE-staining of esophageal tissues at the end of 25th week in different experimental groups (arrow, histological papilloma). **B1-2.** One representative normal histology of esophagus in the control group at 40 × and 100 × magnification, respectively. **B3-4.** One representative esophageal papilloma in the NMBA + Arecoline group at 40 × and 100 × magnification, respectively; C Histological HE-staining of esophageal tissues at the end of 30th week in different experimental groups (arrow, histological papilloma). **C1-2.** One representative normal histology of esophagus in the control group at 40 × and 100 × magnification, respectively. **C3-4**. One representative esophageal papilloma in the NMBA group at 40 × and 100 × magnification, respectively. **C5-6.** One representative esophageal papilloma in the NMBA + Arecoline group at 40 × and 100 × magnification, respectively.

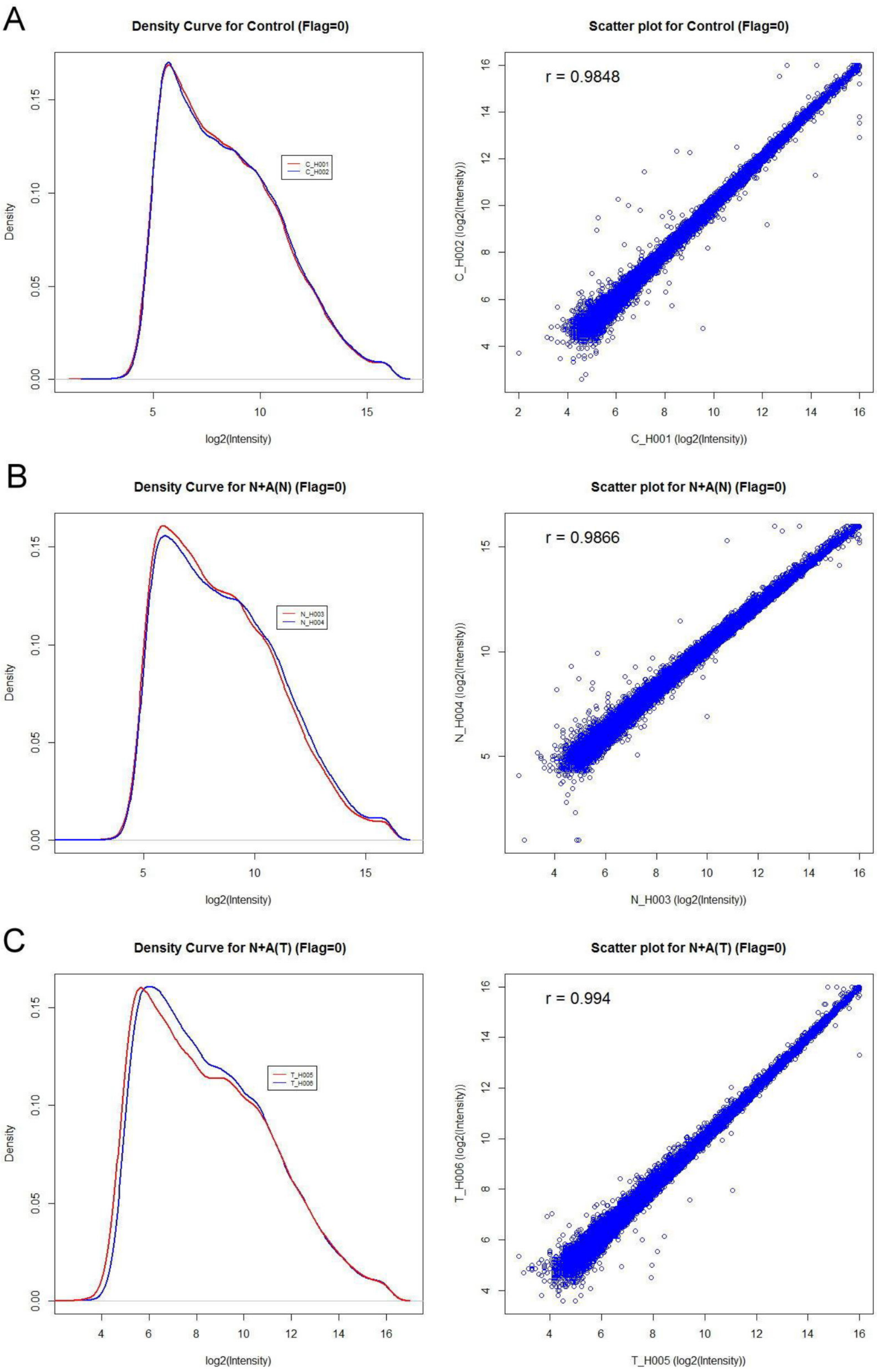

Supplementary Figure S2: The Pearson correlation of gene expression in the repeated arrays of the three pooled tissue samples collected at the end of the 25th week. A. Normal esophageal tissue in the control group; **B.** Normal part of esophageal tissue in the NMBA + Arecoline group; **C.** Tumor part of esophageal tissue in the NMBA + Arecoline group.

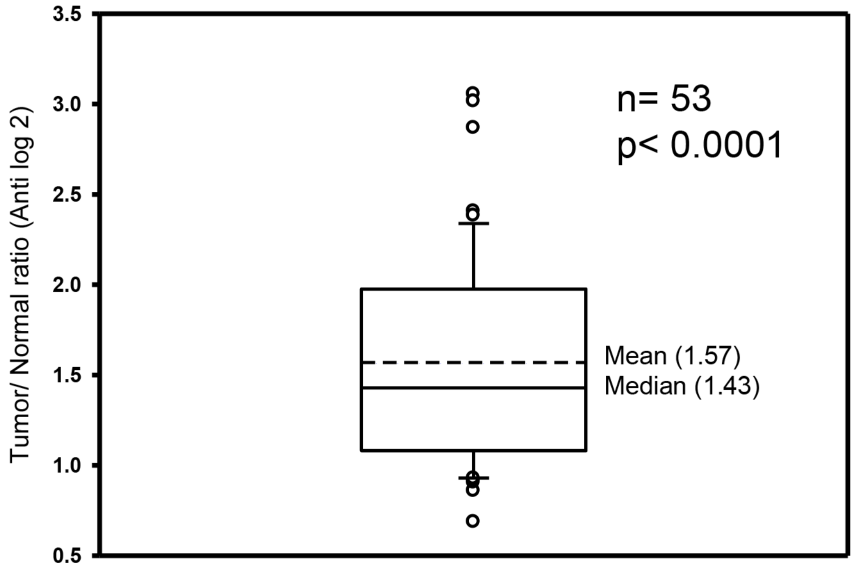

Supplementary Figure S3: ATP1A1 expression in the ratio of tumor/normal parts from 53-paired esophageal squamous cell carcinoma samples (Data modified from Su *et al.*, 2011).

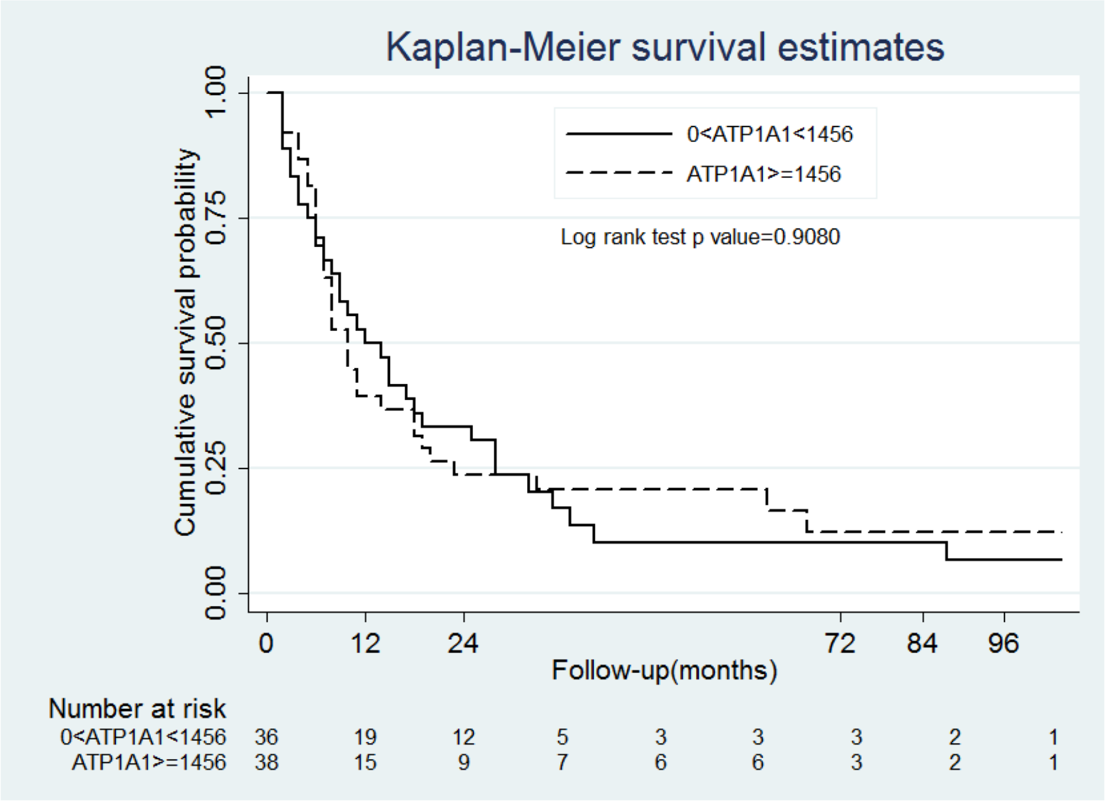

Supplementary Figure S4: Kaplan-Meier survival curve of ESCC patients (N=74) dichotomized by the cut-off level of serum ATP1A1 (1,456 pg/mL).

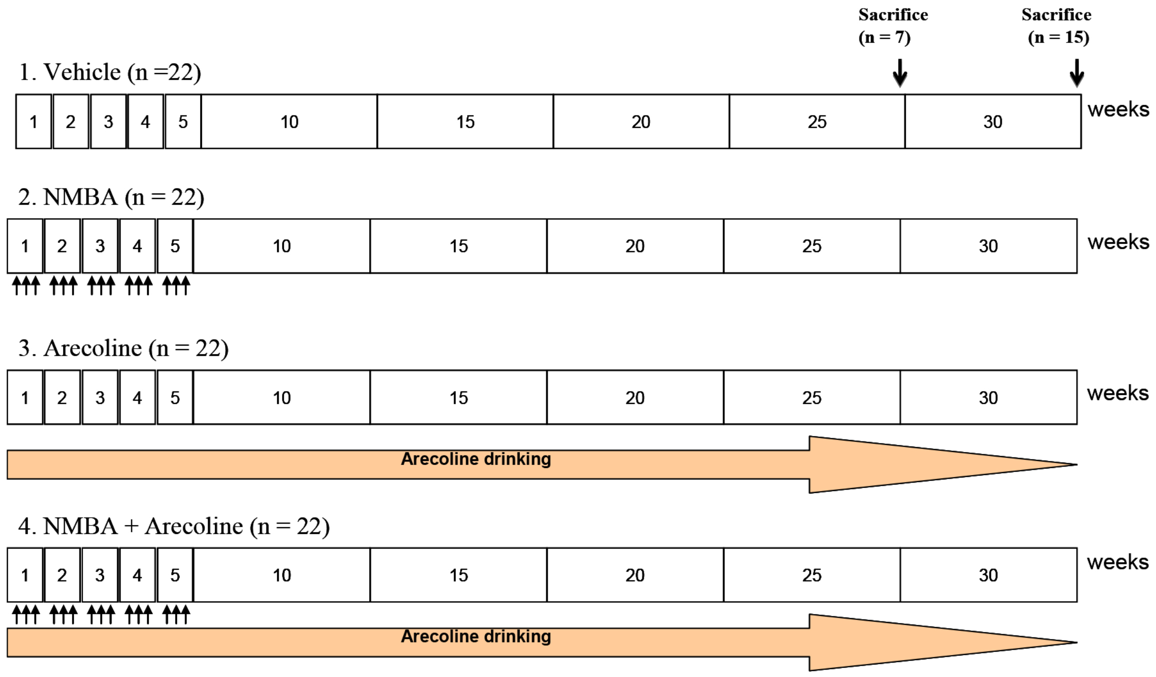

Supplementary Figure S5: Experimental design of rats.

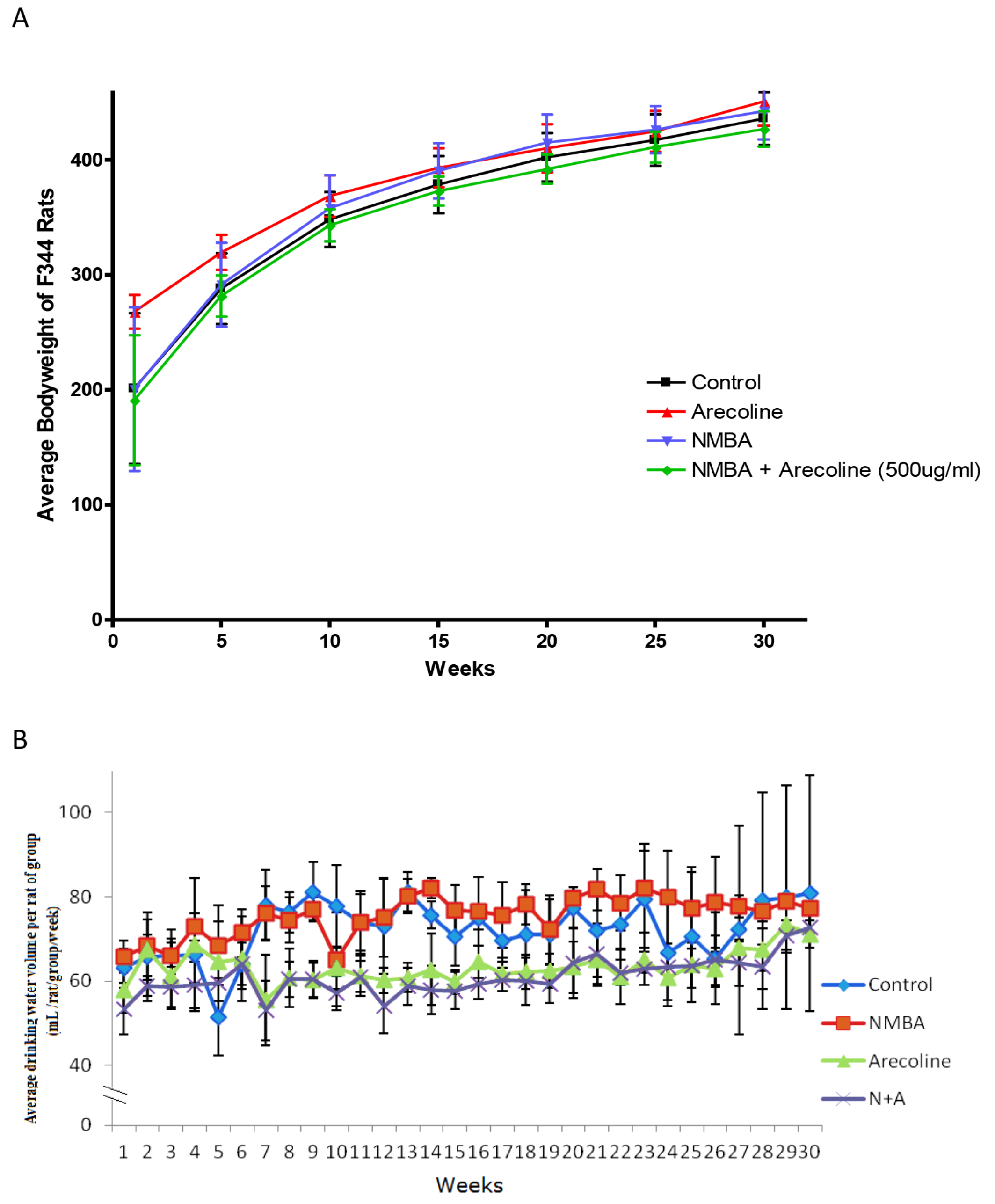

Supplementary Figure S6: Mean growth curve and water consumption in four experimental groups. A. Mean (± standard error (SE)) of growth curve; **B.** Mean (SE) of water consumption in each rat of four groups.

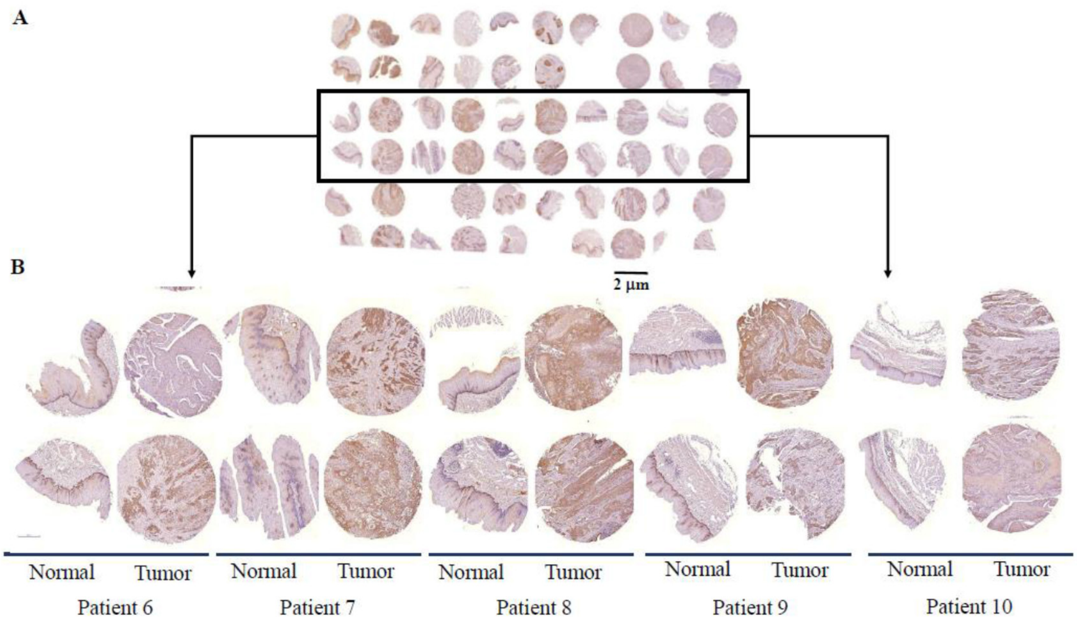

Supplementary Figure S7: ATP1A1 expression in different esophageal tissues by immunohistochemical staining in one representative tissue array slide from Kaohsiung Medical University Hospital, Taiwan. A. Layout of tissue array. One representative slide has 15 case patients. Each case patient has two cancer tissues and two distant normal tissues; **B.** Magnification of one row of tissue array.

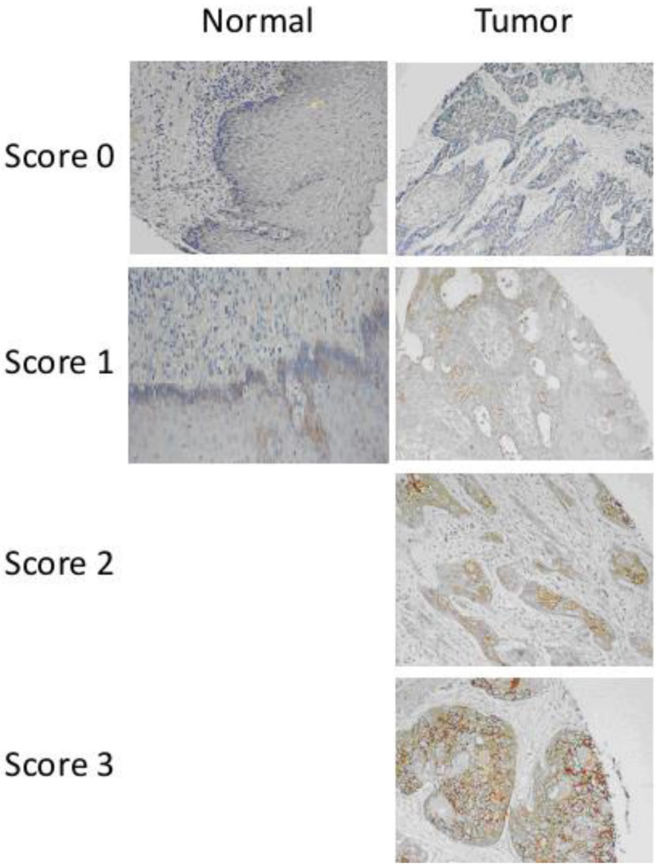

Supplementary Figure S8: Intensity of ATP1A1 immunohistochemical (IHC) staining in esophagus tissue. Representative intensity of ATP1A1 IHC staining in normal and cancer tissues of esophagus (Microscopic magnification ×200).

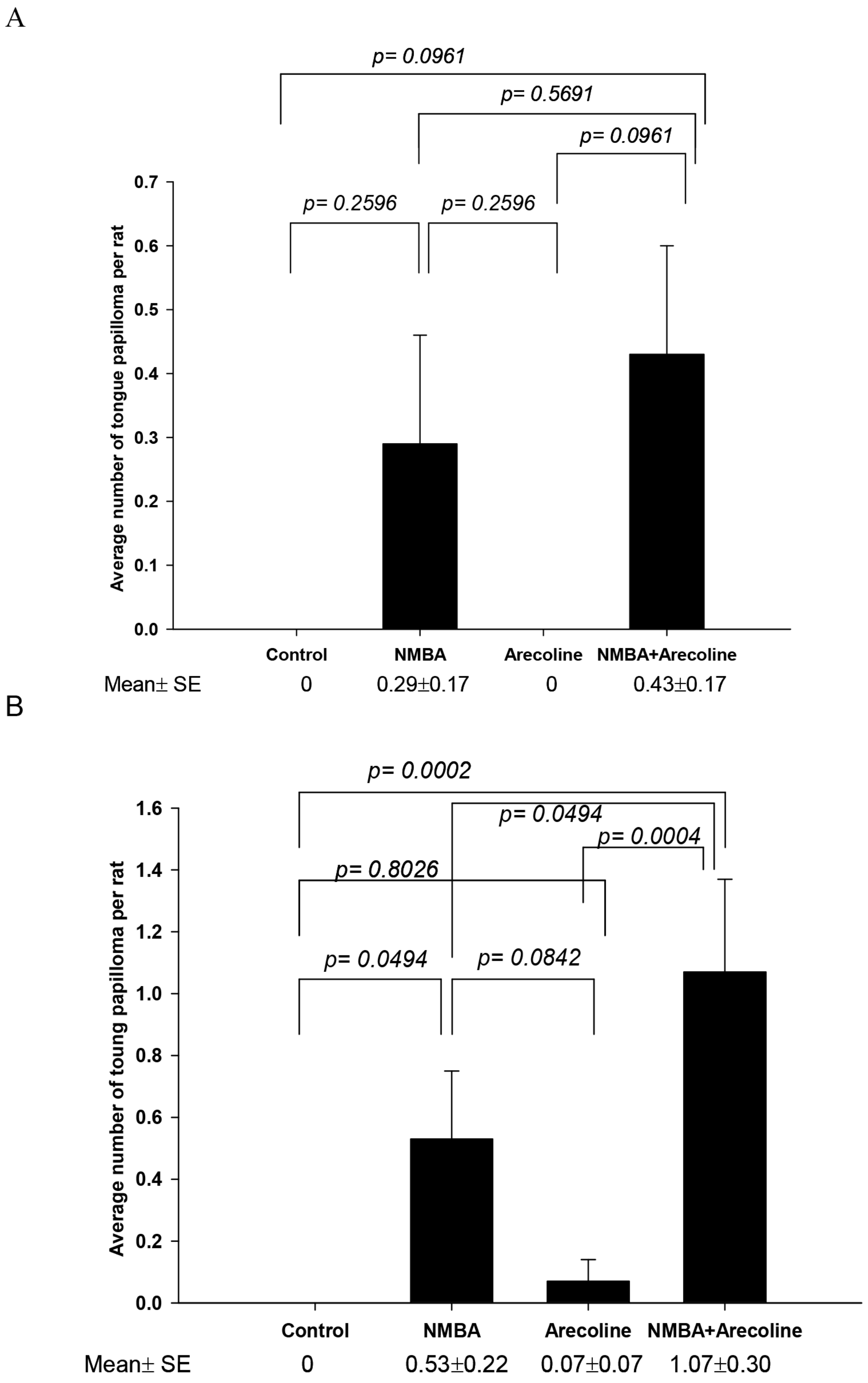

Supplementary Figure S9: Mean (± standard error (SE)) number of tongue papillomas in the four experimental groups. A. Sacrifice in the 25th week (n = 7); **B.** Sacrifice in the 30th week (n = 15).

Supplementary Table S1: Incidence of papillomas in esophagus and tongue of F344 rats sacrificed in the 25th and 30th weeks in four experimental groups

No	Group	Papillomas in the esophagus	
		25th week (N, %)	30th week (N, %)
1	Control	0,0,0,0,0,0,0 (0/7, 0)	0,0,0,0,0,0,0,0,0,0,0,0,0,0,0 (0/15, 0)[2,4]
2	NMBA	0,0,0,0,0,0,0 (0/7, 0)	0,1,0,2,1,1,3,6,0,1,1,0,0,0,0 (8/15, 53.3%)[4]
3	Arecoline	0,0,0,0,0,0,0 (0/7, 0)	0,0,0,0,1,0,0,1,0,0,0,0,0,0,0 (2/15, 13.3%)[3]
4	NMBA + Arecoline	2,1,3,1,2,2,2 (7/7, 100%)[1]	4,5,5,3,5,1,7,0,0,0,0,1,1,1,1 (11/15, 73.3%)[2,3]
**No**	**Group**	**Papillomas in the tongue**	
		**25th weeks (N, %)**	**30th weeks (N, %)**
1	Control	0,0,0,0,0,0,0 (0/7, 0)	0,0,0,0,0,0,0,0,0,0,0,0,0,0,0 (0/15, 0)[5,7]
2	NMBA	0,2,0,0,0,0,0 (1/7, 14.3%)	2,0,0,2,0,1,0,0,0,0,0,1,0,2,0 (5/15, 33.3%)[7]
3	Arecoline	0,0,0,0,0,0,0 (0/7, 0)	0,1,0,0,0,0,0,0,0,0,0,0,0,0,0 (2/15, 6.7%)[6]
4	NMBA + Arecoline	1,1,0,0,1,0,0 (3/7, 42.9%)	1,1,1,1,0,1,0,3,4,2,1,0,0,0,1 (10/15, 66.7%)[5,6]

Fischer exact test.
[1]$p = 0.0006$, when compared with the group of Control, NMBA, or Arecoline.
[2]$p < 0.0001$
[3]$p = 0.0078$
[4]$p = 0.0022$
[5]$p = 0.0002$
[6]$p = 0.0017$
[7]$p = 0.0421$

Supplementary Table S2: The ATP1A1 RNA expression by real-time PCR in 14 ESCC patients and one ESCC cell line.

See Supplementary File 1

Supplementary Table S3: Characteristics of esophageal tumor specimens from three different sources of countries (Taiwan, Korea, and USA)

Country	Slide code	Total patients	ESCC[1]	ESCC+DN[2]	ESCC+AN		
					Control/Case 1[3]	Case 2[4]	Case 3[5]
N		401	369	14	126	243	319
Taiwan	ESCC3-6 (KMUH)	19	16	-	16	-	16
	ESCC11 (CMUH)	29	26	-	26		26
Korea	CR2	60	54	-	-	54	54
USA	ES801	40	39	-	39	-	39
	ES802[6]	40	35	-	-	35	-
	ES1501[6]	75	68	-	-	68	68
	ES2001[7]	120	116	-	30	86	116
	BC02022	18	15	14	15	-	-

Abbreviation: KMUH: Kaohsiung Medical University Hospital; CMUH: China Medical University Hospital Hospital; ESCC = Tumor tissue of esophageal squamous cell carcinoma; ESCC+DN = One tumor part paired with one distant normal part; ESCC+AN: One tumor part paired with one adjacent normal;

[1]Reasons of exclusion: Non-squamous cell carcinoma (n = 5, three adenocarcinoma, one small cell carcinoma, and one basaloid carcinoma); No available tumor part (n = 10); Failure of immunohistochemistry staining of ATP1A1 in tumor part (n = 17).

[2]Available information about ATP1A1 protein expression in both tumor and distant normal parts.

[3]Available information about ATP1A1 protein expression in both tumor and adjacent normal parts.

[4]Available information about ATP1A1 protein expression in tumor only.

[5]Available information about TNM staging.

[6]Duplicated cores per patients.

[7]Seven patients had 2 normal paired cores.

Supplementary Table S4: Distribution of selected demographic and ATP1A1 expression categorized by esophageal cancer patients and control.

See Supplementary File 2

Supplementary Table S5: Mean and median of serum ATP1A1 levels categorized by different clinical characteristics in 78 ESCC patients

ATP1A1 levels[1]				< Median	≥ Median	
	N	Mean ± SD	*p*-value	N (%)		*p*-value
Age (yrs)						
≤ 58	39	1335.3 ± 735.4	0.74	19 (48.7)	20 (51.3)	0.82
>58	39	1390.6 ± 708.2		20 (51.3)	19 (48.7)	
Sex						
Female	5	1429.9 ± 673.0	0.83	2 (40.0)	3 (60.0)	0.64
Male	73	1358.3 ± 724.9		37 (50.7)	36 (49.3)	
Smoking						
No	7	1534.2 ± 580.7	0.51	3 (42.9)	4 (57.1)	069
Yes	71	1346.0 ± 731.1		36 (50.7)	35 (49.3)	
Alcohol						
No	17	1238.0 ± 650.3	0.42	10 (58.8)	7 (41.2)	0.41
Yes	61	1397.7 ± 736.7		29 (47.5)	32 (52.5)	
Betel						
No	36	1281.1 ± 766.0	0.70	20 (55.6)	16 (44.4)	0.36
Yes	42	1433.0 ± 675.0		19 (45.2)	23 (54.8)	
Staging						
I-II	38	1177.7 ± 750.0	0.03	24 (63.2)	14 (36.8)	0.02
III-IV	40	1538.8 ± 646.7		15 (37.5)	25 (62.35)	

Abbreviation: ESCC = Esophageal squamous cell carcinoma; SD = Standard deviation.
[1]Median of ATP1A1 was 1,456 pg/mL.

Supplementary Table S6: Cox regression models in 74 ESCC patients with follow-up more than one month

Variables	Unicariate Cox regression			Multiple Cox regression		
	HR	95% CI		HR	95% CI	
ATP1A1 (pg/mL)						
< 1,456	1			1		
≥1456	0.97	0.60	1.59	0.80	0.45	1.44
Age (years)	1.01	0.98	1.03	1.02	0.99	1.05
Gender						
female	1			1		
male	1.40	0.43	4.50	0.84	0.15	4.81
Cigarette smoking						
No	1			1		
Yes	1.01	0.44	2.35	0.99	0.25	3.91
Alcohol consumption						
No	1			1		
Yes	1.00	0.55	1.81	1.26	0.49	3.20
Betel quid chewing						
No	1			1		
Yes	1.32	0.80	2.17	1.15	0.57	2.31
Stage						
Stage I, II	1			1		
Stage III, IV	2.21	1.33	3.65	2.76	1.48	5.18

Adjusted for all variables listed in this table.

Supplementary Table S7: Clinicopathologic characteristics of 48 esophageal squamous cell carcinoma patients from Kaohsiung Medical University Hospital and China Medical University Hospital, Taiwan

Kaohsiung Medical University Hospital (KMUH)					China Medical University Hospital (CMUH)				
No	Age	Sex	TNM[1]	Stage	No	Age	Sex	TNM[1]	Stage
1	40	Male	T3N0M0	IIA	1	47	Male	T2N0M0	IIA
2	55	Male	T1N0M0	I	2	74	Male	T1N0M0	I
3	62	Male	T3N0M0	IIA	3	37	Male	T2N0M0	IIA
4	62	Male	T1N0M0	I	4	57	Male	T1N0M0	I
5	49	Male	T3N0M0	IIA	5	65	Male	T3N0M0	IIA
6	53	Male	T3N0M0	IIA	6	51	Male	T3N0M0	IIA
7	55	Male	T1N0M0	I	7	52	Male	T3N0M0	IIA
8	60	Male	T2N0M1	IV	8	71	Male	T3N0M0	IIA
9	47	Male	T4N0M0	III	9	42	Male	T3N0M1	IV
10	64	Male	T3N0M1	IV	10	62	Male	T3N0M0	IIA
11	52	Male	T3N0M0	IIA	11	50	Male	T3N0M0	IIA
12	65	Male	T3N0M0	IIA	12	40	Male	T3N0M0	IIA
13	64	Male	T2N0M0	IIA	13	32	Male	T3N0M0	IIA
14	41	Male	T1N0M0	I	14	54	Male	T3N0M0	IIA
15	76	Male	T2N0M0	IIA	15	68	Male	T3N0M0	IIA
16	39	Male	T2N0M0	IIA	16	35	Male	T3N0M1	IV
17	57	Male	T4N0M1	IV	17	57	Male	T3N0M0	IIA
18	60	Male	T3N0M0	IIA	18	55	Male	T3N0M1	IV
19	48	Male	T1N0M0	I	19	47	Male	T4N0M0	III
					20	47	Male	T3N0M0	IIA
					21	57	Male	T3N0M0	IIA
					22	53	Male	T3N0M0	IIA
					23	58	Male	T3N0M1	IV
					24	55	Male	T3N0M0	IIA
					25	44	Male	T3N0M0	IIA
					26	60	Male	T3N0M0	IIA
					27	52	Male	T2N0M0	IIA
					28	41	Male	T2N0M0	IIA
					29	52	Male	T2N0M0	IIA

[1] according to AJCC TNM stage 6th edition
