# Supplementary material for: Overexpression of ATPase Na+/K+ transporting alpha 1 polypeptide, ATP1A1, correlates with clinical diagnosis and progression of esophageal squamous cell carcinoma
Source: Oncotarget. 2016 Nov 10;7(51):85244–58. doi: 10.18632/oncotarget.13267 (PMC5356733; doi:10.18632/oncotarget.13267)
Supplement: Supplementary file 2 [file oncotarget-07-85244-s002.docx]

**Supplementary Table S2. The ATP1A1 RNA expression by real-time PCR in 14 ESCC patients and one ESCC cell line.**

| **Patients** | **Tissues** | **ATP1A1 C_T_** | | **Averaged ATP1A1** | **18S C_T_** | | **Averaged**  **18S** | **Relative ATP1A1 level** | **Gender** | **Age (yrs)** | **Stage**^1^ | **T** | **N** | **M** | **Grade** |
| --- | --- | --- | --- | --- | --- | --- | --- | --- | --- | --- | --- | --- | --- | --- | --- |
| **Pair 1** | **Normal** | 29.242 | 29.046 | 29.144 | 17.409 | 17.586 | 17.4975 | 1 | Male | 42.7 | 3A | 3 | 1 | 0 | 2 |
|  | **Tumor** | 27.372 | 27.881 | 27.6265 | 19.568 | 19.321 | 19.4445 | 11.03871 |  |  |  |  |  |  |  |
| **Pair 2** | **Normal** | 33.889 | 33.99 | 33.9395 | 20.217 | 20.42 | 20.3185 | 1 | Male | 40.4 | 3A | 3 | 0 | 0 | 2 |
|  | **Tumor** | 36.968 | 36.782 | 36.875 | 21.52 | 21.374 | 21.447 | 0.285785 |  |  |  |  |  |  |  |
| **Pair 3** | **Normal** | 28.998 | 28.867 | 28.9325 | 19.975 | 19.897 | 19.936 | 1 | Male | 47.1 | 2A | 3 | 0 | 0 | 2 |
|  | **Tumor** | 29.583 | 29.498 | 29.5405 | 19.804 | 19.566 | 19.685 | 0.551335 |  |  |  |  |  |  |  |
| **Pair 4** | **Normal** | 29.469 | 29.496 | 29.4825 | 19.178 | 19.09 | 19.134 | 1 | Male | 55.9 | 3A | 3 | 2 | 0 | 3 |
|  | **Tumor** | 25.193 | 25.387 | 25.29 | 20.083 | 20.009 | 20.046 | 34.40389 |  |  |  |  |  |  |  |
| **Pair 5** | **Normal** | 29.474 | 29.465 | 29.4695 | 19.558 | 19.301 | 19.4295 | 1 | Male | 60.7 | 3A | 3 | 0 | 0 | 2 |
|  | **Tumor** | 28.916 | 28.708 | 28.812 | 20.958 | 20.967 | 20.9625 | 4.564637 |  |  |  |  |  |  |  |
| **Pair 6** | **Normal** | 29.005 | 28.963 | 28.984 | 18.75 | 18.284 | 18.517 | 1 | Male | 47.7 | 2B | 3 | 0 | 0 | 3 |
|  | **Tumor** | 28.161 | 28.122 | 28.1415 | 19.913 | 19.747 | 19.83 | 4.45523 |  |  |  |  |  |  |  |
| **Pair 7** | **Normal** | 28.379 | 28.188 | 28.2835 | 19.906 | 19.68 | 19.793 | 1 | Male | 46.9 | 2B | 2 | 1 | 0 | 2 |
|  | **Tumor** | 31.415 | 31.326 | 31.3705 | 20.911 | 20.736 | 20.8235 | 0.240399 |  |  |  |  |  |  |  |
| **Pair 8** | **Normal** | 34.422 | 34.595 | 34.5085 | 21.874 | 21.913 | 21.8935 | 1 | Male | 49.7 | 2B | 2 | 1 | 0 | 2 |
|  | **Tumor** | 32.294 | 32.311 | 32.3025 | 21.611 | 21.388 | 21.4995 | 3.511287 |  |  |  |  |  |  |  |
| **Pair 9** | **Normal** | 28.828 | 28.801 | 28.8145 | 19.853 | 19.662 | 19.7575 | 1 | Male | 52.8 | 2B | 3 | 0 | 0 | 2 |
|  | **Tumor** | 28.352 | 28.177 | 28.2645 | 21.468 | 20.987 | 21.2275 | 4.055838 |  |  |  |  |  |  |  |
| **Pair 10** | **Normal** | 29.271 | 29.316 | 29.2935 | 18.671 | 18.781 | 18.726 | 1 | Female | 58.3 | 3A | 3 | 3 | 0 | 2 |
|  | **Tumor** | 32.906 | 32.92 | 32.913 | 21.509 | 21.578 | 21.5435 | 0.573554 |  |  |  |  |  |  |  |
| **Pair 11** | **Normal** | 27.014 | 26.965 | 26.9895 | 19.726 | 18.792 | 19.259 | 1 | Male | 49.1 | 1B | 1a | 0 | 0 | 2 |
|  | **Tumor** | 26.608 | 26.555 | 26.5815 | 20.656 | 20.477 | 20.5665 | 3.284104 |  |  |  |  |  |  |  |
| **Pair 12** | **Normal** | 29.429 | 29.474 | 29.4515 | 19.684 | 19.965 | 19.8245 | 1 | Male | 63.9 | 3A | 3 | 2 | 0 | 2 |
|  | **Tumor** | 27.896 | 28.07 | 27.983 | 20.382 | 20.327 | 20.3545 | 3.995843 |  |  |  |  |  |  |  |
| **Pair 13** | **Normal** | 28.974 | 28.585 | 28.7795 | 14.515 | 14.474 | 14.4945 | 1 | Male | 60.2 | 2A | 2 | 0 | 0 | 2 |
|  | **Tumor** | 27.613 | 27.667 | 27.64 | 14.502 | 14.556 | 14.529 | 2.256364 |  |  |  |  |  |  |  |
| **Pair 14** | **Normal** | 33.232 | 33.323 | 33.2775 | 20.25 | 20.31 | 20.28 | 1 | Male | 64.1 | 3A | 3 | 3 | 0 | 2 |
|  | **Tumor** | 34.54 | 34.34 | 34.44 | 23.25 | 23.23 | 23.24 | 3.476173 |  |  |  |  |  |  |  |
| **ESCC cell line** | | | | | | | | | | | | | | | |
| **CE81T** | | 36.515 | 36.067 | 36.291 | 24.952 | 24.519 | 24.7355 | 1 |  | | | | | | |
| **CE81T1-4** | | 33.978 | 34.032 | 34.005 | 24.829 | 24.983 | 24.906 | 5.660777 |  |  |  |  |  |  |  |

^1^Pathologic stage according to AJCC 7^th^ edition
