# Supplementary material for: Overexpression of ATPase Na+/K+ transporting alpha 1 polypeptide, ATP1A1, correlates with clinical diagnosis and progression of esophageal squamous cell carcinoma
Source: Oncotarget. 2016 Nov 10;7(51):85244–58. doi: 10.18632/oncotarget.13267 (PMC5356733; doi:10.18632/oncotarget.13267)
Supplement: Supplementary file 3 [file oncotarget-07-85244-s003.docx]

**Supplementary Table S4. Distribution of selected demographic and ATP1A1 expression categorized by esophageal cancer patients and control.**

| Variables | ATP1A1 IHC score | | | | | | | | | | | | |
| --- | --- | --- | --- | --- | --- | --- | --- | --- | --- | --- | --- | --- | --- |
|  | Control (n = 126) | | | Case 1 (n = 126) | | | Case 2 (n = 243) | | | Case 3^1^ (n = 319) | | | |
|  | < 1 | ≥ 1 | *p* value | < 1 | ≥ 1 | *p* value | < 1 | ≥ 1 | *p* value | 0-1 | > 1-2 | > 2-3 | *p* value |
| Age (years) |  |  |  |  |  |  | n (%) |  |  |  |  |  |  |
| ≤ 65 | 74 (67) | 37 (33) | 1.00 | 35 (32) | 76 (68) | 0.36 | 27 (14) | 16 (86) | 0.71 | 134 (51) | 79 (30) | 48 (19) | 1.00 |
| > 65 | 10 (67) | 5 (33) |  | 3 (20) | 12 (80) |  | 9 (16) | 46 (84) |  | 27 (47) | 25 (43) | 6 (10) |  |
| Gender |  |  |  |  |  |  |  |  |  |  |  |  |  |
| Male | 57 (59) | 39 (41) | < 0.01 | 25 (26) | 71 (74) | 0.08 | 27 (15) | 156 (85) | 0.96 | 120 (49) | 83 (34) | 43 (17) | 0.29 |
| Female | 27 (90) | 3 (10) |  | 13 (43) | 17 (57) |  | 9 (15) | 51 (85) |  | 41 (56) | 21 (29) | 11 (15) |  |
| Source |  |  |  |  |  |  |  |  |  |  |  |  |  |
| Taiwan | 13 (31) | 29 (69) | < 0.01 | 2 (5) | 40 (95) | < 0.01 | - | - | 0.96 | 14 (33) | 20 (48) | 8 (19) | < 0.01 |
| USA | 71 (85) | 13 (15) |  | 36 (43) | 48 (57) |  | 36 (19) | 153 (81) |  | 138 (62) | 63 (28) | 22 (10) |  |
| Korea | - | - |  | - | - |  | 0 (0) | 54 (100) |  | 9 (17) | 21 (39) | 24 (44) |  |

^1^50 missing data.
